# Evolutionary Adaptations of Plant AGC Kinases: From Light Signaling to Cell Polarity Regulation

**DOI:** 10.3389/fpls.2012.00250

**Published:** 2012-11-16

**Authors:** Eike H. Rademacher, Remko Offringa

**Affiliations:** ^1^Molecular and Developmental Genetics, Institute Biology Leiden, Leiden UniversityLeiden, Netherlands

**Keywords:** AGC kinase, evolution, kinase structure, *Arabidopsis thaliana*, PIN polarity, phototropic growth

## Abstract

Signaling and trafficking over membranes involves a plethora of transmembrane proteins that control the flow of compounds or relay specific signaling events. Next to external cues, internal stimuli can modify the activity or abundance of these proteins at the plasma membrane (PM). One such regulatory mechanism is protein phosphorylation by membrane-associated kinases, several of which are AGC kinases. The AGC kinase family is one of seven kinase families that are conserved in all eukaryotic genomes. In plants evolutionary adaptations introduced specific structural changes within the AGC kinases that most likely allow modulation of kinase activity by external stimuli (e.g., light). Starting from the well-defined structural basis common to all AGC kinases we review the current knowledge on the structure-function relationship in plant AGC kinases. Nine of the 39 *Arabidopsis* AGC kinases have now been shown to be involved in the regulation of auxin transport. In particular, AGC kinase-mediated phosphorylation of the auxin transporters ABCB1 and ABCB19 has been shown to regulate their activity, while auxin transporters of the PIN family are located to different positions at the PM depending on their phosphorylation status, which is a result of counteracting AGC kinase and PP6 phosphatase activities. We therefore focus on regulation of AGC kinase activity in this context. Identified structural adaptations of the involved AGC kinases may provide new insight into AGC kinase functionality and demonstrate their position as central hubs in the cellular network controlling plant development and growth.

## Functional Diversification of Plant AGC Kinases

Cellular responses to external or internal stimuli include the fast modification of the already present machinery of proteins, which might eventually trigger the activation of novel transcriptional programs. A commonly used modification in cellular signaling is protein phosphorylation, which is achieved by the addition of a phosphate group to the side chain of an amino acid. In eukaryotes, protein kinases typically catalyze the transfer of a gamma-phosphoryl group from adenosine triphosphate (ATP) to a serine, threonine, or tyrosine in their substrate proteins. Other amino acids such as histidine, arginine, aspartate, and lysine are uncommon phosphorylation targets in eukaryotes, but often utilized in prokaryotes.

Members of the protein kinase like superfamily have been found in genomes of archae, bacteria, and eukaryotes, highlighting the ancient origin of protein phosphorylation and its evolutionary conservation. With the arrival of multicellular eukaryotic organisms protein phosphorylation has been adopted to control a huge variety of cellular processes, and the involved kinases have evolved to fulfill new tasks, from triggering cell division to regulating membrane transport and cell polarity establishment. Eukaryotic protein kinases (ePKs) have been subdivided into 11 groups based on sequence similarity, evolutionary conservation, and known functions (Hanks and Hunter, 1995; Manning et al., 2002). Six of these subgroups (AGC, CAMK, CKI, CMGC, STE, PKL) are common to 21 eukaryotic genomes covering fungi, animals, plants, apicomplexa, amoebozoa, red algae, and diatoms (Miranda-Saavedra and Barton, 2007).

AGC kinases are among the most well-studied kinases, and are named after the cyclic AMP dependent kinases (PKA), cGMP-dependent kinases, and the diacylglycerol-activated/phospholipid-dependent kinase PKC. In the model plant *Arabidopsis thaliana* the AGC group consists of 39 members (Table 1). The basal member of this group is the 3-phosphoinositide dependent protein kinase 1 (PDK1), which is highly conserved among eukaryotes. Due to its ability to activate other AGC kinases it is considered a master regulator of AGC kinase activity in mammalian cells (Mora et al., 2004). Furthermore, orthologs of the p70 ribosomal protein S6 kinase (S6K), the nuclear Dbf2-related (NDR) kinase subfamily, and the “AGC other” group of mammalian and yeast kinases can be found in *Arabidopsis*. However, the remaining 23 *Arabidopsis* AGC kinases seem to have no counterpart outside the plant kingdom, and are considered a separate subfamily (AGCVIII) in which groups AGC1 to 4 can be distinguished (Hanks and Hunter, 1995; Bögre et al., 2003; Galvan-Ampudia and Offringa, 2007).

**Table 1:** **AGC kinases of *Arabidopsis thaliana***.

Locus	GenPept accession	Names	Full length	N-tail	Activation segment	C-tail	AGC kinase classification	
							1	2
At3g44610	NP_190047.2	AGC1–12	451	77	102	33	AGC1	AGCVIII
At4g26610	NP_194391.1	AGC1–2/D6PKL1	506	131	101	45		
At5g55910	NP_200402.1	AGC1–1/D6PK	498	117	105	47		
At5g47750	NP_199586.1	PK5/D6PKL2	586	199	104	54		
At3g27580	NP_189395.1	PK7/D6PKL3	578	190	102	57		
At2g44830	NP_850426.1	AGC1–3	765	371	102	63		
At5g40030	NP_198819.1	AGC1–4	499	122	98	51		
At1g16440	NP_173094.4	AGC1–6/RSH3	499	121	89	58		
At1g79250	NP_178045.2	AGC1–7	555	154	102	70		
At3g12690	NP_187875.1	AGC1–5	577	193	92	63		
At5g03640	NP_195984.1	AGC1–8	926	549	105	43		
At2g36350	NP_181176.1	AGC1–9	949	567	107	46		
At3g52890	NP_566973.2	KIPK	934	546	109	50		

At2g34650	NP_181012.1	PID	438	83	79	39	AGC3
At2g26700	NP_180238.2	AGC3–4	526	95	137	55	
At1g53700	NP_175774.1	WAG1	476	101	73	71	
At3g14370	NP_188054.1	WAG2	480	96	75	79	

At3g45780	NP_190164.1	PHOT1	996	671	57	39	AGC4
At5g58140	NP_851210.1	PHOT2	915	585	55	46	

At4g13000	NP_193036.1	AGC2–2	372	28	76	45	AGC2
At3g25250	NP_189162.1	AGC2–1/OXI1	421	25	80	87	
At1g51170	NP_564584.1	AGC2–3/UCN	404	30	83	59	
At3g20830	NP_188719.1	AGC2–4/UCNL	408	29	85	62	

At4g14350	NP_193171.2	NDR-1	551	127	69	134		AGCVII
At1g03920	NP_171888.1	NDR-2	569	145	68	129		
At3g23310	NP_188973.2	NDR-3	568	128	71	148		
At2g19400	NP_565453.1	NDR-4	527	113	77	114		
At2g20470	NP_179637.2	NDR-5	569	132	67	143		
At4g33080	NP_195034.2	NDR-6	519	102	77	112		
At1g30640	NP_174352.1	NDR-7	562	128	73	140		
At5g09890	NP_568221.1	NDR-8	515	110	72	112		

At5g62310	NP_201037.1	IRE	1168	762	59	120		AGC other
At1g48490	NP_564529.4	IRE-3	1235	836	53	119		
At1g45160	NP_175130.2	IRE-4	1042	678	55	82		
At3g17850	NP_188412.2	IRE-H1	1296	890	59	120		

At3g08720	NP_187485.1	S6K1	465	142	27	70		AGCVI
At3g08730	NP_187484.1	S6K2	471	148	27	71		

At5g04510	NP_568138.1	PDK1–1	491	50	38	174		PDK1
At3g10540	NP_187665.2	PDK1–2	486	51	38	168		

*The table represents all 39 AGC kinases found in *Arabidopsis*. The first three columns give the TAIR locus and GenPept accession numbers for each kinase as well as some of the names assigned to the genes, respectively. The fourth column represents the full length of the respective amino acid sequence and is followed by the length of the N-tail (counted until the second glycine of the conserved GxGxxG motive in subdomain I), the length of the activation segment (DFD/GLS to APE), and the length of the C-tail (counted from the tryptophan of the FxxxxW motive in subdomain XI). The last two columns represent the classification of *Arabidopsis* AGC kinases 1) as proposed by Galvan-Ampudia and Offringa (2007), 2) based on earlier classifications (Hanks and Hunter, 1995; Bögre et al., 2003)*.

Based on amino acid sequence homology these plant specific AGCVIII kinases are most closely related to animal PKA and PKC, which are involved in the regulation of cell polarity, -growth, and -division, and for which no direct homologs are found in plants. Plant AGCVIII kinases have evolved into regulators of a variety of developmental processes and stress responses. In unicellular green algae, such as *Chlorella variabilis*, *Ostreococcus tauri*, and *Chlamydomonas reinhardtii*, orthologs of the *Arabidopsis* PHOTOTROPIN 2 (PHOT2) are the only AGCVIII kinases that can be identified, indicating that these are the direct descendants of the first ancestral AGCVIII protein kinase (Onodera et al., 2005; Derelle et al., 2006; Galvan-Ampudia and Offringa, 2007; Blanc et al., 2010). This implies that the ancestral plant AGCVIII kinases acquired an N-terminal regulatory photoreceptor domain that was lost in later descendants. Interestingly, phot2 in land plants is involved in responses to high light intensity, such as light avoidance of chloroplasts and actin-dependent positioning of the nucleus (Iwabuchi et al., 2010), which are typically functions that are required in unicellular plant life. Later in evolution, a second phototropin (PHOT1) co-occurred with the first appearance of seed plants (Galvan-Ampudia and Offringa, 2007). Phot1 mediates responses to low intensity blue light (BL) and exclusively mediates BL-dependent inhibition of hypocotyl elongation of germinating seeds reaching the substrate surface (Folta and Spalding, 2001). Combined these two AGC4 kinases enable seed plants to optimize their photosynthetic efficiency and subsequent growth in response to changing light conditions.

Closely related but distinct from the other AGCVIII kinases are the four AGC2 kinases OXIDATIVE SIGNAL-INDUCIBLE1 (OXI1/AGC2–1), AGC2–2, UNICORN (UCN/AGC2–3), and UNICORN-LIKE (UCNL/AGC2–4). OXI1 and AGC2–2 have been shown to be involved in root growth (Anthony et al., 2004; Camehl et al., 2011), oxidative stress signaling (Rentel et al., 2004), and plant defense responses (Petersen et al., 2009). A recent report indicated that UCN and UCNL regulate cell growth and division in integuments, the embryo proper, cotyledons, and floral organs (Enugutti et al., 2012).

The remaining 17 AGCVIII kinases can be subdivided in two groups, AGC1 and AGC3 (Galvan-Ampudia and Offringa, 2007). Of the AGC3 kinases PINOID (PID), WAG1, and WAG2 have been shown to regulate the polarity of auxin transport by phosphorylating the large central hydrophilic loop (HL) of PIN-FORMED (PIN) auxin efflux carriers (Michniewicz et al., 2007; Dhonukshe et al., 2010; Huang et al., 2010). The role of the fourth kinase AGC3-4/PID2 is still ambiguous, as based on the current data it is still unclear whether this kinase acts redundantly with the other three AGC3 kinases during embryo development (Cheng et al., 2008; Dhonukshe et al., 2010). Also four of the AGC1 kinases (D6 PROTEIN KINASE (D6PK)/AGC1–1, D6 PROTEIN KINASE LIKE 1 (D6PKL1)/AGC1–2, D6PKL2/PK5, D6PKL3/PK7) have been implicated to have a regulatory role in polar auxin transport. Although they can phosphorylate the PIN-HL *in vitro*, their exact function is still unclear (Zourelidou et al., 2009). AGC1–5 and AGC1–7 have been implied in polarized pollen tube growth (Zhang et al., 2009). To our knowledge the role of the other 7 *Arabidopsis* AGC1 kinases has not been determined so far, except that KCB INTERACTING PROTEIN KINASE (KIPK) was found to interact with a kinesin-like protein (Day et al., 2000). In tomato, the AGC1 kinase AvrPto-DEPENDENT Pto-INTERACTING PROTEIN 3 (Adi3) has been implied as possible ortholog of mammalian Akt/PKB, as both proteins act as negative regulator of programmed cell death (PCD; Devarenne et al., 2006). Based on homology the putative negative regulator of PCD in *Arabidopsis* would be AGC1–3.

## Structural Features of Plant AGC Kinases

Misregulation of AGC kinase activity in mammalian systems causes the development of severe diseases including cancer and diabetes. Naturally, this has attracted intense research and has led to the elucidation of various crystal structures of human and mammalian AGC kinases as well as the fine mapping of relevant subdomain functions (Taylor and Kornev, 2011).

Of the *Arabidopsis* AGC kinases only phot1 and phot2 have been studied in structural detail, with particular emphasis on the photosensory domains. A recently published structure of a phot2 fragment comprising the kinase domain and one of the photosensory domains remains the only available plant AGC kinase structure to date (Takayama et al., 2011). The structure of phot2 underlines that evolutionary conservation of structural elements can be found within the group of AGC kinases. Hence, it should be possible to draw conclusions on the structure and regulation of plant AGC kinases based on what is known from well-researched animal AGC kinases.

### The catalytical core of AGC kinases

**Figure 1:**
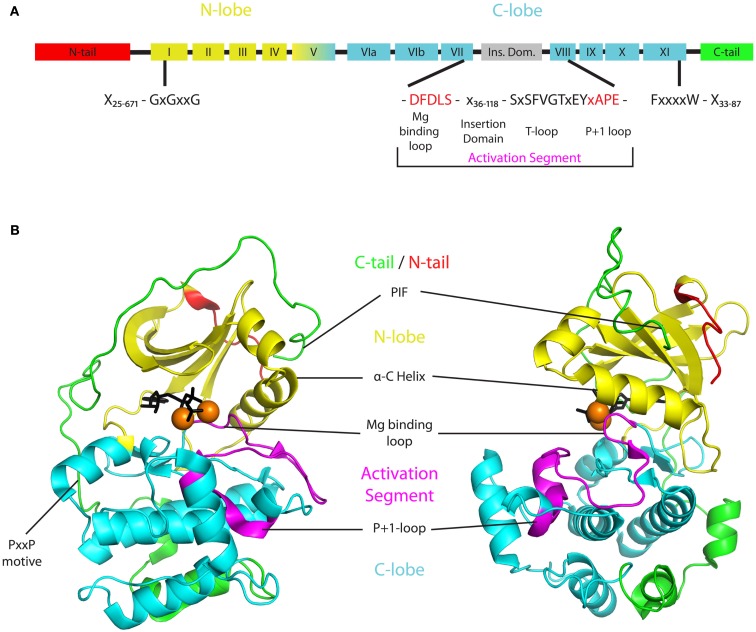
**Basic structure of AGCVIII kinases**. **(A)** Two dimensional structure indicating the different protein domains of AGCVIII kinases. The N- and C-lobe (yellow or cyan) of the catalytic core are supplemented by regulatory domains in the N-tail (red), activation segment (magenta), and C-tail (green). These three domains are highly variable and their minimal and maximal lengths in AGCVIII kinases are denoted [N-tail 25–671 amino acids (AA), activation segment 55–137 AA, C-tail 33–87 AA]. Importantly, the magnesium binding loop (DFDLS) and the activation loop (SxSFVGTxEY) are separated by a 36–118 AA long insertion domain that is found in plant AGC kinases only. **(B)** Exemplary three dimensional structure of an AGC kinase with the same color coding as in the two dimensional structure. The depicted structure is based on a ternary crystal structure of mouse PKA [PDB-ID: 4DG2 (Bastidas et al., 2012)]. The beta-sheet rich N-lobe (yellow) is connected to the alpha-helix rich C-lobe (cyan) by a linker formed by subdomain V. Together they form the catalytic core of AGC kinases and enable ATP (black), magnesium (orange spheres), and substrate (not shown) binding in the catalytic cleft between the lobes. The activity of AGC kinases is regulated by post-translational modification of residues in, or binding of interactors to the N-tail (red), activation segment (magenta), or C-tail (green). The N-tail depicted here represents the anchor of a, in some cases much bigger, regulatory domain that extends from the core structure. Also the activation segment and the C-tail are highly diverse. The activation segment starts with a magnesium binding loop (DFDLS, black) and ends with the *P* + 1 loop (APE, purple) that is involved in substrate binding. Different from the depicted situation in PKA, a quite variable insertion domain of up to 118 AA can be found within the activation segment of plant AGC kinases. Likewise, the C-tail of most plant AGC kinases contains conserved elements, such as the PxxP motif or the PIF at its C-terminus (green), but also shows great diversity in composition and size.

Like all protein kinases, plant AGC kinases contain the universal catalytic core that is built up of 12 conserved subdomains (Figure 1A; Hanks and Hunter, 1995). This catalytic core binds ATP together with Mg^2+^ and catalyzes the transfer of a phosphate group onto a substrate protein/amino acid. It is formed by two lobes that are interconnected by a linker domain (Figure 1). The smaller N-terminal lobe (subdomains I–IV) is dominated by five β-strands but also features a prominent conserved α-helix, called α-C helix (subdomain III). The larger and more rigid C-terminal lobe on the other hand is mainly formed by α-helices (subdomains VIa–XI). The flexible linker domain between the two lobes (subdomain V) functions as a hinge and allows for rotation of the lobes and subsequent opening or closure of the ATP binding pocket located in the cleft between them (Figure 1B). Correct positioning of a highly conserved glycine-rich loop (GxGxxG, P-loop) in subdomain I of the N-lobe and an invariant aspartate in subdomain VII of the C-lobe is mandatory for ATP binding in the catalytic cleft. Furthermore subdomains VIB and VIII within the C-lobe form the binding sites for protein substrates (Hanks and Hunter, 1995; Nolen et al., 2004; Taylor and Kornev, 2011). Accordingly, binding of ATP and substrates is regulated by conformational changes in the catalytic core. These changes are evoked by phosphorylation of accessory domains protruding from the core at the N- or C-terminus (called N- or C-terminal tail), and of the activation segment that comprises the residues between two conserved tripeptides (DFG and APE) found in respectively subdomains VII and VIII of the C-lobe.

### Regulatory domains in the N-terminal tail

The N-terminal tail (N-tail) is highly variable between AGC kinases and extends over the N-lobe. While the N-tail of the PDK1s and AGC2 kinases is relatively short (25–50 amino acids) it is relatively large in several other AGC kinases (up to 890 amino acids in IREH-1; Table 1). This makes it likely that the N-tail is a site for post-translational regulation of kinase activity, either by different modifications such as myristoylation, deamidation, and phosphorylation, or by binding of regulatory cofactors. Even though such modifications and interactions have been described for mammalian PKA and Akt/PKB (Currie et al., 1999; Tholey et al., 2001; Sastri et al., 2005; Bastidas et al., 2012), functional studies on plant AGC N-tails are largely lacking. Only the N-tails of PHOT1 and PHOT2 have been analyzed in greater detail and photosensory domains as well as various phosphorylation sites have been mapped (for review see (Christie, 2007)).

### The multifunctional activation segment is extended in plant AGC kinases

The activation segment of protein kinases generally contains a magnesium binding loop (DFG), a T-loop (also called activation loop), and a *P* + 1 loop (xAPE, Figure 1A; Nolen et al., 2004; Taylor and Kornev, 2011). The latter forms a critical interaction point between substrate and kinase, while the aspartate in the DFG motif of the magnesium binding loop is responsible for chelating one of the Mg^2+^-ions that orient the ATP for phospho-transfer. The following phenylalanine contributes to the formation of the kinase – substrate contact surface by interacting with residues of the N-lobe (Nolen et al., 2004). While these two residues are invariant in all AGC kinases, the glycine in the DFG motif has been exchanged for an aspartate in plant AGCVIII kinases. This amino acid change appears to reduce kinase activity *in vitro* (Christensen et al., 2000).

In AGC kinases, phosphorylation of a serine or threonine residue in the T-loop ([**S/T**]**^P^**xxGTx[D/E]Y) is necessary to activate the kinase (Chan et al., 1999; Bögre et al., 2003). Phosphorylation of the T-loop results in a conformational change involving the α-C helix (Figure 1B) that stabilizes the kinase in an open structure allowing substrate binding (Huse and Kuriyan, 2002). Vice versa, when the T-loop is in an inactive conformation a preceding beta-sheet becomes disordered and can no longer anchor the activation segment in the catalytic cleft. Subsequently, binding of Mg^2+^ and substrate peptides is inhibited (Nolen et al., 2004).

Interestingly, in the plant specific AGCVIII kinases a larger insertion of 36–118 amino acids in the activation segment, also referred to as the T-loop extension (Bögre et al., 2003), VII–VIII insertion (Galvan-Ampudia and Offringa, 2007), or insertion domain (Zegzouti et al., 2006b), separates the Mg^2+^ binding site (DFDLS) and the T-loop (Sx**S^P^**FVGTxEY; Figure 1A). This insertion domain is typical for plant AGC kinases, and for some of the AGC kinases it was found to determine their subcellular localization. The insertion domain of *Arabidopsis* PID kinase has been shown to direct its plasma membrane (PM) association (Zegzouti et al., 2006b), and for the tomato AGC kinase Adi3 it contains a nuclear localization sequence (NLS) (Ek-Ramos et al., 2010). For other kinases the function of the insertion domain is unclear, but it is likely that it provides an additional structural feature to the kinase that allows further regulation of its activity or its subcellular localization.

In most of the ACGVIII kinases the insertion domain contains a conserved AEP motif directly linked to the T-loop. The function of this AEP motif is not clear, but the fact that it can also be found in PHOT2 orthologs in unicellular algae, confirms that AGCVIII kinases are direct descendants from an ancestral phototropin. Its absence in the AGC2 kinases suggests that this group branched off at a certain point in evolution from the other AGCVIII kinases by losing this feature. It will be interesting to see if the AEP motif is a hallmark for the functional divergence between these two groups of plant AGC kinases.

### The C-terminal tail provides protein-protein interaction sites

Other than the N-tail and the activation segment that both emerge from the catalytic core and form relatively independent subunits, the C-terminal tail (C-tail) stretches from the C-lobe around the kinase core and eventually folds back on to the N-lobe (Figure 1B). Three conserved segments have been identified within the C-tail that are shared by most AGC kinases. These are the C-lobe tether, the active-site tether, and the N-lobe tether (Kannan et al., 2007). All three have been implicated to be involved in mediating the opening and closing of kinases by interacting with conserved residues in the catalytic core. Regulatory motifs in these segments might serve as interaction sites for other factors. For example, a conserved PxxP motif in the C-lobe tether of mammalian Akt/PKB has been shown to serve as a binding site for its activating tyrosine kinase Src (Jiang and Qiu, 2003). Furthermore, a hydrophobic motive (FxxF) at the C-terminus of the N-lobe tether was found to mediate interaction with PDK1 and was therefore named PDK1 interacting fragment (PIF, Figure 1B; Etchebehere et al., 1997; Biondi et al., 2000; Frodin et al., 2002). In some AGC kinases this motif is extended by a phosphorylation site (FxxF[S/T]Y]), the phosphorylation of which enhances PDK1 binding and hence allows for an additional level of regulation (reviewed in Biondi, 2004). Next to mediating PDK1 interaction, *in vitro* reconstitution assays suggest that the PIF acts synergistically with T-loop phosphorylation in stimulating kinase activity. Potentially it does so by stabilizing the αC helix in an active conformation that allows for optimal transfer of the ATP phosphate group to the substrate (Frodin et al., 2002).

## Regulation of Plant AGC Kinases Activity

The activity of protein kinases is regulated by posttranslational modification and by interacting proteins or macromolecules, both of which have an effect on their enzymatic activity and/or on their subcellular localization. The latter in turn determines the proximity to and thus the chance to phosphorylate their downstream targets. Most of these regulatory aspects involve the N- or C-terminal tail and the activation segment of the protein kinase. For the plant AGCVIII kinases, considerable research has been done on the BL receptors phot1 and phot2, and events leading to their activation are relatively well understood. Due to their role in regulating polar auxin transport, also PID, and two other AGC3 protein kinases (WAG1 and WAG2) have been analyzed in more detail. Compared to the phototropins, however, crystal structures and a detailed investigation of regulatory domains of the AGC3 protein kinases are missing. Below we will therefore summarize the current knowledge, and speculate on possible regulatory pathways with a focus on the AGC3 kinases and their role in PIN trafficking.

### PDK1 as regulator of AGC kinase activity

As indicated in Section “The Multifunctional Activation Segment is Extended in Plant AGC Kinases,” AGC kinases require phosphorylation of the T-loop for their activation. In mammalian systems several AGC kinases are phosphorylated by PDK1 that acts as a master regulator of AGC kinase activity. Other AGC kinases, including PDK1, are able to auto-activate through cis- or trans-autophosphorylation.

For PDK1, however, cis- and trans-autophosphorylation of its T-loop is not sufficient for activation, since it is initially auto-inhibited by its pleckstrin homology (PH) domain. This phosphoinositide binding domain bends over the catalytic cleft and blocks docking of protein substrates (Casamayor et al., 1999; Wick et al., 2003; Gao and Harris, 2006). In mammalian cells such “primed” but inactive PDK1 molecules reside in the cytoplasm as homo-dimers, interacting via the PH domain, ATP loaded, and with the T-loop phosphorylated. Binding of the PH domain to 3-phosphoinositides in the PM leads to dissociation into monomers and stimulates trans-autophosphorylation of a threonine close to the PH domain. This may stabilize an open conformation of the activated PDK1 monomers, which are the active PM-associated forms that interact with and phosphorylate target AGC kinases, such as Akt/PKB (Wick et al., 2003; Masters et al., 2010).

PDK1 interacts with the hydrophobic motif in the PIF domain of its substrate kinases (Figure 1B) through a PIF binding pocket present in its N-lobe (Biondi et al., 2000, 2001). Human AGC kinases often have a serine or threonine residue directly following their PIF domain (FxxF/Y**[S/T]^P^**F/Y) that needs to be phosphorylated before interaction with PDK1 can occur. In the mammalian AGC kinases PKCζ and PKC related kinase 2 (PRK2) an aspartic acid and glutamic acid residue are respectively present at the serine/threonine position, and these residues are required for binding of and activation by PDK1, probably because they mimic the negative charge of a phosphorylated serine/threonine (Balendran et al., 2000).

Two *Arabidopsis* homologs of mammalian PDK1 (PDK1–1 and PDK1–2) have been identified. Based on the presence of a PIF domain in several plant AGC kinases, as well as on protein–protein interaction studies and *in vitro* activity assays, *Arabidopsis* PDK1 potentially is involved in T-loop phosphorylation and activation of at least 16 AGC kinases (Bögre et al., 2003; Anthony et al., 2004; Zegzouti et al., 2006a,b). Interestingly, most of these AGC kinases terminate in the tetrameric hydrophobic motif (FxxF) of the PIF domain and lack an adjacent serine/threonine residue.

Exceptions to this are the PIFs of AGC1–12 and S6K1 and 2. In the case of AGC1–12 the FxxF motif is hidden approximately 90 amino acids upstream of the C-terminus. In S6K1 and 2 the FxxF motif is found 15 amino acids upstream of the C-terminus. Recently it has been shown that the hydrophobic motif of the S6Ks is expanded to a TARGET OF RAPAMYCIN (TOR) phosphorylation motif of the form FxxF**T**^P^YVxP, and that this motif is phosphorylated by TOR kinase in *Arabidopsis* (Xiong and Sheen, 2012). Analogous to mammalian systems *Arabidopsis* TOR has been demonstrated to interact with S6K1 via REGULATORY-ASSOCIATED PROTEIN OF TOR (RAPTOR). Osmotic stress leads to removal of RAPTOR and the subsequent loss of PIF domain phosphorylation by TOR, which inhibits PDK1 dependent activation of S6K1 (Mahfouz et al., 2006).

Recently, doubts were raised, based on different observations, as to whether PDK1 is a master regulator of the plant specific AGCVIII kinases (Zhang and McCormick, 2009). A similar discussion is ongoing for the PKA isoforms, the closest animal homologs of the AGCVIII kinases. *E. coli* expressed PKA has been reported to auto-phosphorylate *in vitro* (Yonemoto et al., 1997), but other reports suggest that activation of PKA requires the activity of PDK1 (Cheng et al., 1998; Nirula et al., 2006). Interestingly, like the plant AGCVIII kinases, PKA also has a tetrameric FSEF motif at its C-terminus. It is thus tempting to speculate that kinases with the tetrameric C-terminal hydrophobic motif are less or even not dependent on PDK1 for their activation. In line with this hypothesis, PID:GUS or PID:VENUS translational fusions in which the FDYF motif was excluded were able to complement the *pid* loss-of-function mutant (Benjamins et al., 2001; Michniewicz et al., 2007). Although it has been shown *in vitro* that the PIF domain is essential for a functional interaction with PDK1 (Zegzouti et al., 2006a), the relevance of this domain and of PDK1 for the *in planta* function of PID still needs confirmation. PID can activate itself through intramolecular autophosphorylation, which may be sufficient *in planta* under optimal growth conditions. The *in vitro* observed 6.5-fold increase in PID phosphorylation in the presence of PDK1 may have a function in stressed plants (Zegzouti et al., 2006a). Also the close PID homologs WAG1 and WAG2 are able to auto-phosphorylate, despite the fact that they do not have a C-terminal PIF domain and are not hyperactivated by PDK1 *in vitro*. Unexpectedly, they do interact with PDK1 in *in vitro* pull downs, and addition of the FDYF motif to the C-terminus of WAG1 enhances its autophosphorylation, whereas the presence of PDK1 reduces its activity (Zegzouti et al., 2006b). In line with this observation, the autophosphorylating AGC1 kinase KIPK has a FxxF motif and interacts with PDK1 but is not hyperactivated by PDK1 *in vitro* (Zegzouti et al., 2006b). All these data suggest that the interaction with PDK1 is not necessarily mediated by the FxxF motif, and that other residues may be important.

The *Arabidopsis* phototropins PHOT1 and PHOT2 lack a PIF domain (Bögre et al., 2003) and like WAG1 and WAG2 seem to act independently of PDK1. Both have been shown to auto-phosphorylate their N-terminal photosensory domain and their T-loop in response to BL (Salomon et al., 2003; Inoue et al., 2008). This action is controlled by two accessory light, oxygen, or voltage sensing (LOV) domains at the N-terminus of the kinases, of which LOV2 under dark conditions represses kinase activity by folding over the catalytic core. The LOV domains bind flavinmononucleotides (FMNs) as chromophores to sense BL, which induces the formation of a covalent adduct between FMN and a cystein residue in each LOV domain (Salomon et al., 2000; Swartz et al., 2001). This results in dissociation of the LOV2 domain from the kinase domain, and leads to receptor dimerization and subsequent trans-phosphorylation as shown for phot1 (Kaiserli et al., 2009).

### Dynamic localization of AGC kinases

Apart from being activated, protein kinases need to be brought into proximity of their substrates. Vice versa, separation of the kinase from its substrates by subcellular re-localization is an efficient mechanism to regulate kinase activity. AGC protein kinases phosphorylate nuclear, cytosolic, or membrane localized target proteins, and their proper localization relies on internal localization signals or on binding to scaffold proteins, or specific membrane components.

An example of re-localization in animal kinases is the current model for the previously discussed Akt/PKB activation by PDK1. PDK1 can change from a cytosolic homodimer into a monomer that binds Akt/PKB. Association of this heterodimer through the PH domains of both proteins to the PM triggers PDK1 to phosphorylate Akt/PKB, and the subsequent release of active Akt/PKB. The monomeric PDK1 then dissociates from the membrane to form a “primed” homodimer, or to retrieve the next Akt/PKB target protein (Masters et al., 2010).

**Figure 2:**
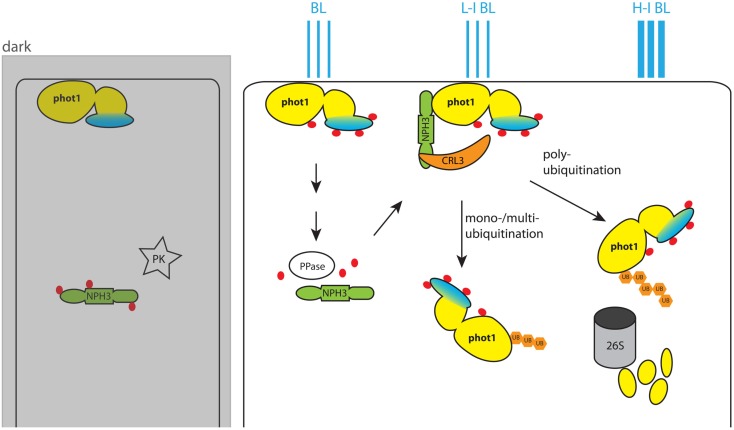
**Model for blue light (BL)-dependent regulation of phot1 activity**. In the dark phot1 is unphosphorylated and associates to the PM. NPH3 on the other hand is phosphorylated by an unknown protein kinase (PK) and remains in the cytosol. BL triggers autophosphorylation of phot1, and this leads to dephosphorylation of NPH3 by an unidentified phot1-controlled protein phosphatase. Dephosphorylated NPH3 heterodimerizes with CUL3 and forms the CRL3^NPH3^ E3 ubiquitin ligase. In this ligase NPH3 functions as anchor for the PHOT1 substrate. In low intensity BL (L-I BL) the activity of CRL3^NPH3^ leads to mono-/multiubiquitination of PHOT1 and its subsequent localization to the cytoplasm. With higher BL intensities (H-I BL) CRL3^NPH3^ mediates polyubiquitination of phot1. This marks phot1 for degradation by the 26S proteasome and eventually desensitizes the plant cell for BL by reducing the amount of available receptors. The phot1 kinase domain is colored yellow, and the photoreceptor domain is marked in blue. Phosphate groups on the proteins are indicated by red dots, while ubiquitin groups are represented by orange hexagons.

In plants the activity of the phototropins seems also to be regulated by re-localization. In dark grown seedlings phot1 and 2 localize to the PM. A pulse of BL induces partial endocytosis of phot1 (Sakamoto and Briggs, 2002) and association of phot2 with the Golgi apparatus (Kong et al., 2006). Recent work suggests that internalization of phot1 is regulated by the ubiquitination status of the kinase (Roberts et al., 2011), the mechanism of which will be further discussed below (see Section “Plant AGC Kinase Activity Dynamics in Development and Growth”, Figure 2).

Similarly, investigation of a complementing *PID::VENUS* fusion revealed that PID is mainly present at the PM, close to its phosphorylation targets the PIN proteins (Michniewicz et al., 2007). That PM localization is important for PID action was indicated by the reduced effect of PID overexpression in a sterol biosynthesis mutant background (Dhonukshe et al., 2010). In this mutant background PID:VENUS was found to localize predominantly to the cytosol, indicating that the membrane composition is important for PID PM localization, and that PID PM localization is important for PID function. PID can also be found in punctuate structures in the cytoplasm (Michniewicz et al., 2007; Dhonukshe et al., 2010), which is intriguing, since the only demonstrated function of PID is phosphorylation of PIN family members at the PM. It is tempting to speculate that these cytoplasmic structures are either part of a desensitization mechanism and involved in PID turn-over, or that PID has other phosphorylation targets in the cytosol.

In maize the PID co-ortholog BARREN INFLORESCENCE2 (BIF2) has been identified. Like PID, a YFP:BIF2 fusion localizes to the PM and phosphorylates the HL of ZmPIN1a *in vitro* (Skirpan et al., 2008, 2009). This is necessary for proper localization of ZmPIN1a in developing inflorescence meristems (Skirpan et al., 2009), and suggests that phosphorylation-directed control of PIN protein targeting by AGC kinases is a conserved regulatory mechanism, at least in seed plants. Localization studies in onion epidermis and *Nicotiana benthamiana* cells have revealed that YFP:BIF2 is also present in the nucleus, where it seems to interact with and to phosphorylate the basic helix-loop-helix transcription factor BARREN STALK1 (BA1). Nuclear localization of BIF2 might be mediated through its interaction with BA1 or through a yet unidentified nuclear localization signal (Skirpan et al., 2008). Nuclear localization has not been reported for PID, however, two other AGC3 kinases WAG1 and WAG2 seem to act similar to BIF2, in that they phosphorylate PINs at the PM (Dhonukshe et al., 2010) and localize to the nucleus (Galvan-Ampudia and Offringa, 2007) where they possibly regulate the activity of nuclear proteins.

The actual protein domains involved in PM association of phot1/2 and PID are not known. A transmembrane domain is clearly absent in these proteins, implying that PM association is achieved by interaction with other PM-bound proteins or through binding to lipid components of the PM. Binding of PID to several phosphoinositides and phosphatidic acid (PA) in a lipid-overlay assay suggested that the latter is true. A fusion of GFP with the insertion domain of PID localizes to the PM in yeast cells, demonstrating that this domain is sufficient for PM localization. This is further substantiated by the redirection of AGC1–7 from the cytoplasm to the PM in tobacco cells after exchange of its insertion domain for the PID insertion domain (Zegzouti et al., 2006b). Despite these indications, mechanisms that regulate subcellular localization of PID and the other AGC3 kinases remain elusive.

*Arabidopsis* PDK1 was also found to bind to phosphoinositides and PA in lipid-overlay assays through a PH domain present at its C-terminus (Deak et al., 1999). Importantly, only binding to PA stimulates PDK1 activity in a kinase assay (Anthony et al., 2004; Otterhag et al., 2006), suggesting that PDK1 activity might be restricted to specific (PA-containing) locations at the PM.

### Regulation by interacting proteins and second messengers

Apart from regulation of AGC protein kinase activity by other kinases and re-localization of the kinases and their substrates, only a few other regulatory factors have been identified.

Mammalian PDK1 is negatively regulated by the 14-3-3 protein θ. A similar role has been indicated for 14-3-3 proteins in *Arabidopsis* by assaying the phosphorylation of S6K2 by PDK1 in the presence of 12 14-3-3 isoforms. In this assay autophosphorylation of PDK1 and subsequent phosphorylation of S6K2 was enhanced by 9 14-3-3s (μ, Φ, κ, ω, ε, ψ, υ, χ, ν) and completely inhibited by another (o; Otterhag et al., 2006). Since in all cases phosphorylation levels of PDK1 were changed by the 14-3-3 proteins, it was suggested that the 14-3-3 proteins facilitate dimerization of PDK1 and that this enhances the activating trans-autophosphorylation of PDK1. Recent reports also indicate a role for 14-3-3 proteins in the regulation of phot1 and 2. An interaction between phot1 and the 14-3-3 proteins λ, κ, Φ, υ was demonstrated in yeast-two-hybrid assays and by immuno-precipitation from plant extracts (Inoue et al., 2008; Sullivan et al., 2009). Interestingly, the interaction of phot1 with 14-3-3 λ was found to be light dependent in both assays, but a role for this interaction was not investigated (Sullivan et al., 2009). Phot2 on the other hand was found to interact with 14-3-3 λ in a yeast-two-hybrid assay. Further investigation of 14-3-3 λ binding to fragments of phot2 demonstrated that the kinase domain is sufficient for this interaction. Furthermore, replacement of a serine in the 14-3-3 binding site (RSK[S→A]QP) located in the T-loop of PHOT2 was sufficient to abolish this interaction in yeast. *In planta* loss of 14-3-3 λ reduces phot2 functionality only in BL induced stomatal opening, while other phot2 regulated processes are not affected (Tseng et al., 2012). In conclusion, 14-3-3 proteins seem to interact with AGC kinases and to influence their activity. Whether this is by enhancing protein interactions for trans-autophosphorylation or substrate phosphorylation, or by stabilizing the active conformation of AGC kinases has yet to be determined.

Together with interacting proteins, second messengers play an essential role in the activation of several AGC kinases. Earlier we described the dependence of PDK1 activation on interaction with 3-phosphoinositides at the PM (see Sections “PDK1 as Regulator of AGC Kinase Activity” and “Dynamic Localization of AGC Kinases”). In a similar manner binding of cyclic guanosine monophosphate (cGMP) is necessary for the activation of isoforms of the cyclic nucleotide-dependent kinase PKG. Here, binding of cGMP initiates conformational changes that relieve the kinase from an autoinhibitory domain. Isoforms of PKA, protein kinase X (PRKX), and PRKY are controlled by cyclic adenosine monophosphate (cAMP), but in the off state these kinases exist as hetero-tetramers of two catalytic (C-) and two regulatory (R-) subunits. When G-protein coupled receptors activate adenylate cylase, the produced cAMP binds to the R subunits, causing dissociation of the C subunits, and activation by phosphorylation of their activation loop (reviewed in Pearce et al., 2010). Even though cAMP and cGMP based signaling has been shown to be important in plant development and stress responses, the existence of kinases that respond to concentration changes of these second messengers remains elusive (Isner et al., 2012).

Another well-known mechanism is calcium-dependent regulation of protein kinase activity. A plant- and protozoan-specific class of calcium-regulated kinases are the Calmodulin-like Domain Protein Kinases (CDPKs) that have their own calmodulin (CaM) domain with four calcium binding pockets (EF-hands) to sense the calcium concentration. Binding of Ca^2+^ to the CaM domain relieves the repression by the autoinhibitory junction domain and thus leads to kinase activation (Hrabak et al., 2003). Other kinases only have a CaM binding domain and are therefore named CaM dependent protein kinases (CaMKs). CaMKs are activated by CaM binding, and are found in animals, but have only occasionally been identified in plants (Carafoli et al., 2001). A plant-specific group of CaMKs that has been initially identified in lily is the calcium and CaM-dependent protein kinase (CCaMK; Hrabak et al., 2003). Besides a CaM binding domain, CCaMKs have a visinin-like C-terminal domain with three EF-hands. A well-studied example of the CCaMKs is DMI3, which plays a central role in the symbiotic interaction of legumes with rhyzobia and arbuscular mycorrhizal fungi. In both cases DMI3 translates the micro-organism-induced calcium spiking into respectively root nodule and arbuscular mycorrhiza formation. The EF-hands in the C-terminal domain seem to regulate kinase autophosphorylation at basal calcium levels, which in turn enhances binding of the CaM at calcium peaks, relieving auto-inhibition, and leading to activation of the kinase and substrate phosphorylation (Sathyanarayanan et al., 2000; Gleason et al., 2006; Swainsbury et al., 2012). CaM-dependent kinases are only found in certain plant species, and neither CaMK, nor CCaMK can be found in *Arabidopsis* (Hrabak et al., 2003).

Regulation by Ca^2+^ has been reported for the conventional animal PKC isoforms. Calcium binding to the C2 domain enhances PM association and subsequent activation of PKCs (Newton, 2001). To our knowledge regulation of AGC kinases through direct binding of CaMs has not been reported in animal systems. In contrast, the *Arabidopsis* PID kinase activity is regulated by the small calcium binding protein PID-BINDING PROTEIN 1 (PBP1) and the CaM-like protein TOUCH3 (TCH3). Binding of these proteins to PID is enhanced by calcium. However, while PBP1 interacts weakly with PID in the absence of Ca^2+^, binding of TCH3 is strictly Ca^2+^-dependent. Importantly, autophosphorylation of PID is enhanced by addition of PBP1 in the kinase assay, while it is repressed by TCH3 (Benjamins et al., 2003). There is some analogy in the regulation of PID activity by two types of calcium binding proteins and the calcium-dependent regulation of the CCaMK DMI3 (Swainsbury et al., 2012), which leads to the interesting hypothesis that PBP1 and TCH3 might allow modulation of PID kinase activity in response to oscillating cytosolic calcium levels.

Previously, it was reported that PID is directly inhibited *in vitro* by 15 mM Ca^2+^. Because PID does not have an obvious Ca^2+^ binding pocket, and co-incubation with 15 mM Ca^2+^ and Mg^2+^ restores PID activity, this inhibition is likely to be a result from competitive replacement of the Mg^2+^ ion in the catalytic core by Ca^2+^ (Zegzouti et al., 2006a). Hence, it might be that AGC kinase activity is regulated by the availability of mandatory cofactors such as Mg^2+^ and ATP. The chance, however, that Ca^2+^ will be inhibiting PID *in vivo* is low, as reported cytosolic Mg^2+^ concentrations are in the millimolar range, whereas those for Ca^2+^ range from sub-micromolar to micromolar (Hepler, 2005).

Finally, evidence for direct binding of the natural kinase inhibitor quercetin to PID was recently provided (Henrichs et al., 2012). Other than the kinase inhibitors staurosporine and chelerythrine, the flavonol quercetin is able to efficiently inhibit substrate phosphorylation by PID. This coincides with the reported function of quercetin as a natural polar auxin transport inhibitor (Jacobs and Rubery, 1988). Both PID expression and flavonoid biosynthesis are upregulated in response to auxin, PID expression within 2 h, and flavonoid accumulation only 8–12 h after auxin treatment (Benjamins et al., 2001; Lewis et al., 2011). The flavonol quercitin could thus provide feedback control on auxin enhanced PID kinase activity.

## Plant AGC Kinase Activity Dynamics in Development and Growth

In the previous sections we have described important structural features of plant specific AGC protein kinases as well as known regulatory events that directly act on these protein kinases to modulate their activity. While this might help in understanding AGC protein kinase functionality, many regulatory pathways that have not been directly connected to the kinases had to be left out. Here we will use the modulation of auxin responses and transport by on the one hand the most ancestral AGCVIII kinases phot1 and phot2, and on the other hand the land plant specific AGCVIII kinases PID, WAG1, and WAG2 to provide examples of how all aspects of AGC kinases are integrated in their role as regulators of plant development.

### Regulating phototropic growth

Phot1 and phot2 regulate various processes in response to BL. Both kinases have been shown to regulate phototropism, stomatal opening, chloroplast accumulation movement, and cotyledon/leaf flattening. Additionally, a role of both proteins in regulating leaf movement has been implicated. Next to these shared roles in which both phototropins only distinguish themselves by acting at respectively lower and higher light fluence rates, they also have unique regulatory roles. Phot1 has been shown to induce hypocotyl growth inhibition and to be involved in the regulation of mRNA stability, whereas chloroplast relocation to avoid intense light solely depends on phot2 (Christie, 2007).

For all these processes various factors were identified that act downstream of phot1/phot2-mediated phosphorylation. Yet apart from BL of different intensities, only a few mechanisms were indicated in fine tuning the activity of one or both phototropins. As mentioned earlier, phototropins locate to the PM in dark grown seedlings and are relocated to cytoplasmic- or Golgi-like structures after BL treatment (Sakamoto and Briggs, 2002; Kong et al., 2006). For phot1, internalization has been suggested to be modulated by ubiquitination through the CULLIN-RING E3 ubiquitin ligase CRL3^NPH3^ to which it binds via the substrate adopter NON-PHOTOTROPIC HYPOCOTYL 3 (NPH3) (Roberts et al., 2011). Interestingly, in dark grown seedlings NPH3 is found to be phosphorylated. Yet a BL pulse leads to its dephosphorylation in a phot1-dependent manner (Pedmale and Liscum, 2007). Neither the kinase that phosphorylates NPH3 in the dark nor the protein phosphatase that dephosphorylates it in response to phot1 activation have been identified to date (Figure 2). Possibly, dephosphorylation enhances the affinity of NPH3 for phot1, and at the same time stabilizes the assembly of CRL3^NPH3^. Roberts and coworkers propose a model in which the degree of phot1 ubiquitination is depending on BL intensity (Figure 2): under low intensity BL phot1 would be mono-/multi-ubiquitinated and potentially internalized into endosomes via a clathrin-dependent mechanism (Kaiserli et al., 2009; Roberts et al., 2011). Higher BL intensities would lead to polyubiquitination of phot1 and its subsequent recruitment for degradation by the 26S proteasome, thereby desensitizing cells for BL (Roberts et al., 2011).

A phot2:GFP fusion was shown to interact with the PM through its kinase domain, and to co-localize with a Golgi marker in a punctate pattern in the cytosol few minutes after its activation by BL treatment (Kong et al., 2006). A strikingly similar punctate pattern was observed for the osmotic stress-related protein kinase SnRK2.4 a few minutes after application of salt stress (McLoughlin et al., 2012). It is tempting to speculate that these are similar subcellular structures involved in rapid feed back regulation of kinase activity. *Chlamydomonas* phot2 can complement the *Arabidopsis* mutant (Onodera et al., 2005), and its activation was proposed to lead to a conformational change of the insertion domain (Pfeifer et al., 2010). If we assume that, analogous to PID, the insertion domain is responsible for PM association, this conformational change might thus be responsible for dissociation or trafficking from the PM.

Next to NPH3, various other proteins have been shown to interact with the phototropins (for review see Inoue et al., 2010). Of these only ATP BINDING CASSETTE B19 (ABCB19) and recently PHYTOCHROME KINASE SUBSTRATE 4 have been identified as substrate of phot1 (Christie et al., 2011; Demarsy et al., 2012).

In protein extracts from 3-day-old etiolated seedlings phosphorylated PKS4 (PKS4L) could be detected after 30 s of BL irradiation. A maximum of PKS4L was detectable after 10 min of irradiation, after which PKS4L levels decreased and finally were no longer detectable after 4 h of irradiation. PKS4L was detectable in *phot2* mutants but absent in mutants lacking phot1 or expressing a kinase dead versions of it. In addition, a truncated version of PHOT1 was found to phosphorylate several fragments of PKS4 *in vitro*. This demonstrates that PKS4 is a substrate of phot1. Accordingly, accumulation of PKS4L could be enhanced either by irradiation with BL of higher intensities, or by treatment with a phosphatase inhibitor. Measurement of phot1-mediated phototropic bending in response to BL indicated that unphosphorylated PKS4 positively regulates phototropism, while this response is inhibited by PKS4L (Demarsy et al., 2012). Although PKS4 is likely to be part of a negative feedback loop on phot1 activity, a direct effect of this protein on phot1 activity remains to be demonstrated.

**Figure 3:**
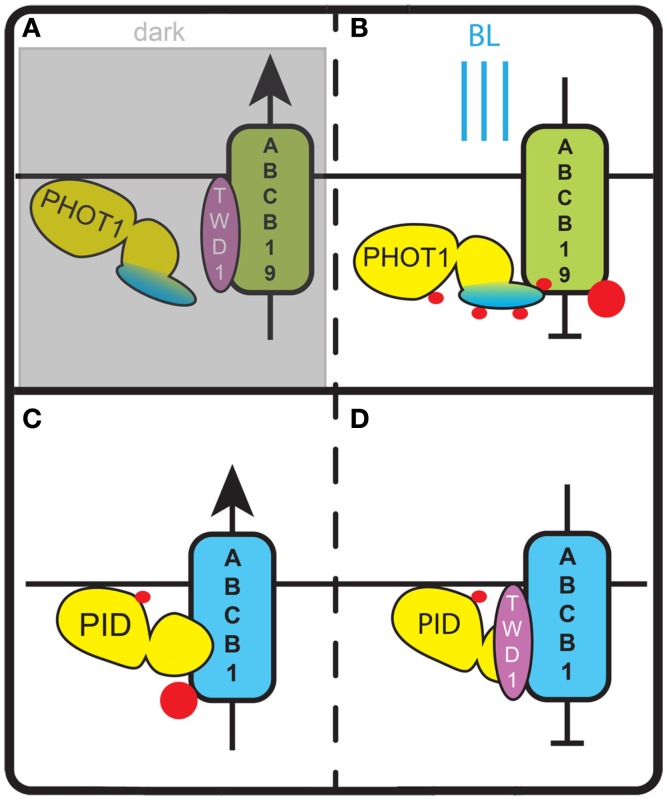
**Regulation of ABCB auxin transporters by AGC kinases**. **(A)** In darkness phot1 is inactive and does not phosphorylate ABCB19. In this state ABCB19 can interact with its positive modulator TWD1 and export auxin from the cell. **(B)** Blue light (BL) activates phot1 and induces phosphorylation of ABCB19. This inhibits ABCB19 – TWD1 interaction and blocks auxin efflux via ABCB19. **(C)** In the absence of TWD1, phosphorylation of a serine in the linker domain of ABCB1 by PID stimulates ABCB1 activity and leads to an increase of auxin export. **(D)** In the presence of TWD1, PID is no longer able to phosphorylate the linker domain of ABCB1. This lack of phosphorylation and potentially the phosphorylation of other sites of ABCB1 block ABCB1 activity and the respective auxin efflux. Red dots indicate phosphorylation events.

ABCB19 is an auxin efflux carrier that functions in the long distance transport of auxin from the shoot apical meristem to the root, and the immunophilin TWISTED DWARF1 (TWD1) has been suggested to act as a positive modulator of its activity (Bouchard et al., 2006). Binding of ABCB19 and its phosphorylation by phot1 was demonstrated *in vitro* and in yeast cells. In HeLa cells co-transfected with ABCB19 and phot1 auxin efflux is abolished after BL treatment, indicating that phosphorylation of ABCB19 by phot1 inhibits the auxin transporter. Further immuno-precipitation assays showed that the interaction between TWD1 and ABCB19 is lost after BL treatment of wildtype seedlings, but can be detected in equally treated *phot1* seedlings. This indicates that phot1-dependent phosphorylation of ABCB19 disrupts the positive modulation of ABCB19 by TWD1 at the light side of the hypocotyl (Christie et al., 2011; Figures 3A,B). The subsequent PIN mediated redirection of auxin flow toward the shaded hypocotyl side (Ding et al., 2011) would stimulate cell expansion on that side and thus result in phototropic bending in response to a directional BL stimulus.

### Regulating polar auxin transport

**Figure 4:**
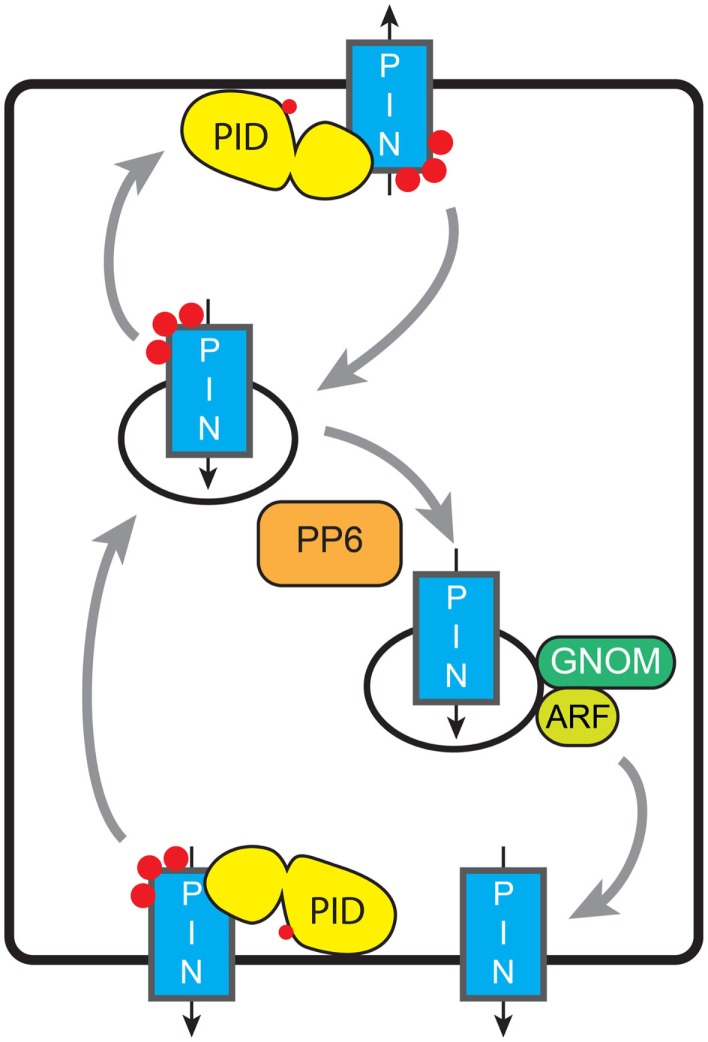
**Model for phosphorylation-dependent re-localization of PINs**. De novo synthesized PINs are distributed apolarly to the PM. Constitutive endocytosis relocates PINs depending on their phosphorylation status (red dots). This relocation might be baso-apical as in the figure (embryo, root, shoot, and inflorescence meristem), apolar-lateral (phototropism), between lobe and indentation (pavement cells), or between the inner or outer cell side (guard cells). Tuning of the activities of AGC kinases and PP6-type phosphatase determines the PIN phosphorylation status and thus the preferential targeting pathway, and leads to the respective redistribution of the PINs. Basal targeting of unphosphorylated PINs is GNOM-dependent; ARF-GEFs involved in other targeting pathways remain unknown. Grey arrows indicate trafficking of PIN-loaded vesicles, while black arrows indicate the direction of PIN-mediated auxin transport.

The studies on the phototropins have revealed how their activity is regulated by light. A few components in the respective downstream signaling pathways, such as for example NPH3 (Roberts et al., 2011), PKS4 (Lariguet et al., 2006), and ABCB19 (Christie et al., 2011), have been uncovered. However, the signaling processes downstream of phototropins are only now beginning to be unraveled. In contrast, studies on the redundantly acting *Arabidopsis* AGC3 kinases PID, WAG1, and WAG2 have revealed the PIN auxin efflux carriers as one of their main targets (Michniewicz et al., 2007; Dhonukshe et al., 2010; Huang et al., 2010), while the exact functions of identified proteins that bind to PID or its maize ortholog BIF2 remain unclear (Benjamins et al., 2003; Skirpan et al., 2008). The three *Arabidopsis* AGC3 kinases were shown to phosphorylate serine residues in three conserved TPRxS[N/S] motifs in the HL of PIN1-type auxin efflux carriers, leading to apical (shootward) PIN localization, whereas loss-of-phosphorylation was shown to induce a PIN polarity shift toward the basal (rootward) cell membrane (Friml et al., 2004; Dhonukshe et al., 2010; Huang et al., 2010). The three AGC3 kinases act antagonistically in determining PIN polarity with a novel PP6-type heterotrimeric protein phosphatase, consisting of a PP2AA regulatory subunit, the PP6 catalytic subunit FyPP1 or FyPP3, and the SAPS DOMAIN-LIKE protein SAL that binds to the PIN-HL in a phosphorylation-dependent manner (Michniewicz et al., 2007; Dai et al., 2012; Figure 4). Interestingly, several other serine and threonine residues in the PIN-HL have been identified as *in vivo* phosphorylation targets (Nuhse et al., 2004; Benschop et al., 2007; Michniewicz et al., 2007; Chen et al., 2010). For at least two of these residues in the PIN1-HL it was shown that their phosphorylation status determines the asymmetric distribution of PIN1 (Zhang et al., 2010). It is likely that these other residues are phosphorylated by other kinases that remain to be identified. Potential candidates are the four D6 and D6-like protein kinases of the AGC1 group (AGC1-1/D6PK, AGC1-2/D6PKL1, PK5/D6PKL2, PK7/D6PKL3). These kinases were shown to affect auxin transport, possibly by phosphorylating the PIN-HL, but their activity does not lead to changes in PIN polarity (Zourelidou et al., 2009; Dhonukshe et al., 2010). It will be interesting to see whether these kinases recognize the same or different residues in the PIN-HL, or that they act on auxin transport via a different mechanism.

An important question that remains to be answered is: what in the cell recognizes the phosphorylation status of PIN proteins to direct their proper localization at the PM? Studies with PIN–fluorescent protein fusions indicate that *de novo* synthesized PIN proteins are initially placed on the PM in an apolar manner, where most likely their phosphorylation status is determined by the competing activities of PM-associated AGC3 kinases and PP6-type phosphatases (Dhonukshe et al., 2010). Following clathrin- and sterol-dependent endocytosis (Dhonukshe et al., 2007; Men et al., 2008), the PIN-HL phosphorylation status is somehow sensed, resulting in recycling to the PM at the correct side of the cell. This dynamic trafficking of PIN-loaded vesicles has been shown to involve the actin cytoskeleton (Geldner et al., 2001), and the action of ADP Ribosylation Factors (ARFs; Xu and Scheres, 2005), their downstream Rho GTPases (ROPs; Xu et al., 2010; Nagawa et al., 2012) and their upstream ARF GTP exchange factors (ARF-GEFs; Geldner et al., 2001, 2003; Sieburth et al., 2006; Kleine-Vehn et al., 2008). Long term inhibition of the ARF-GEF GNOM by low concentrations of the fungal toxin brefeldin A (BFA) was also found to lead to a basal-to-apical transcytosis of PIN proteins, indicating that GNOM is involved in recycling of PIN proteins to the basal PM, whereas other ARF-GEFs induce trafficking to the apical membrane (Geldner et al., 2003; Kleine-Vehn et al., 2008). The phosphorylation status of the PIN-HL was shown not to inhibit PIN endocytosis (Dhonukshe et al., 2010), but rather to determine the recruitment of PIN proteins into the GNOM-independent trafficking pathway (Kleine-Vehn et al., 2009). The phosphorylation signal thus directs super-polar PIN targeting during recycling, whereas maintenance of PIN polarity by recruitment of PINs into non-mobile PM clusters and by spatially defined endocytosis (Kleine-Vehn et al., 2011) seems to be independent of the PIN phosphorylation status. AGC3 kinase-mediated shifts in PIN polarity have been shown to be important for tropic growth responses (Sukumar et al., 2009; Dhonukshe et al., 2010; Ding et al., 2011), and for patterning during embryogenesis and organ formation at the shoot and inflorescence meristems (Kleine-Vehn et al., 2009; Huang et al., 2010; Li et al., 2011). However, not for all processes changes in AGC3 kinase activity lead to the predicted apico-basal shift in PIN localization. For example, the phototropic response of the hypocotyl of etiolated *Arabidopsis* seedlings was shown to be mediated by BL dependent relocation of the PIN3 auxin efflux carrier (based on a complementing *PIN3::PIN3:GFP* fusion) from a symmetric distribution to an asymmetric localization at the inner lateral side of hypocotyl endodermis cells. Genetic evidence was provided that at least the PID kinase is involved in this response. The observed phot1-dependent down regulation of *PID* expression was proposed to lead to PIN3 loss-of-phosphorylation and thus to GNOM dependent trafficking of PIN3 proteins to the inner lateral side (Ding et al., 2011). How the phototropin signal is translocated to the nucleus to affect PID transcription, and whether the phot-dependent rapid increase in cytosolic Ca^2+^ concentration following BL stimulation (Baum et al., 1999; Babourina et al., 2002; Harada et al., 2003) has a role in these responses is unclear. The possibility that the phot1 kinase directly phosphorylates PIN proteins was excluded by *in vitro* phosphorylation reactions (Ding et al., 2011), indicating that phototropins and AGC3 kinases have different substrate specificities.

The PID/PP6-dependent phosphorylation status of PIN1 was also shown to modulate the interdigitated pattern of epidermis cells in *Arabidopsis* leaves. PID overexpression or PP6 loss-of-function induced a shift of PIN1:GFP localization to the indentations, leading to a reduction in the degree of pavement cell indentation, whereas phosphatase overexpression reversed this to wildtype PIN1:GFP localization in the lobe (Li et al., 2011). PID overexpression or PP6 loss-of-function were also shown to induce re-localization of PIN1:GFP from the inner to the outer side of stomatal guard cells (Li et al., 2011). In conclusion, depending on the cell- and tissue type there are variations on the initial paradigm that AGC3 kinase-mediated phosphorylation of PIN proteins directs their baso-apical polarity. The central message is that *de novo* synthesized PIN proteins come into contact with these kinases upon arrival at the PM, and that phosphorylation leads to their sorting in the GNOM-independent pathway, whether this is apical in meristem epidermis cells, lateral-outer in hypocotyl endodermis cells, or outer in stomatal guard cells. The PM association of PID close to its phosphorylation targets is central here, and it will be interesting to map the site that allows association with the PM. In addition it will be important to establish how the calcium binding PID interacting proteins affect PID activity in response to changes in the cytosolic calcium concentration.

A recent study provides evidence that PID does not only phosphorylate PIN auxin efflux carriers, but also acts on an auxin transporter of the ABCB family (Figures 3C,D). ABCB auxin transporters are predominantly apolarly localized in plant cells and have been proposed to reduce net-influx of auxin from the apoplast (Mravec et al., 2008). Evidence was obtained that PID modulates the auxin transport activity of ABCB1 by phosphorylating a serine residue in the linker domain of this transporter (Henrichs et al., 2012). It is tempting to speculate that this occurs equivalent to the modulation of ABCB19 activity by phot1, and that accordingly ABCB1 phosphorylation by PID disrupts the interaction with TWD1 (Figure 3). However, measurements of auxin efflux from *Nicotiana benthamiana* protoplasts co-transfected with PID and ABCB1 showed increased efflux, whereas co-transfection of ABCB1 and TWD1 led to a reduction in efflux. Co-transfection of PID, ABCB1, and TWD1 in the same system completely abolished auxin efflux. In yeast cells, the contradictory finding that ABCB1 activity is reduced by PID co-expression might be explained by a yeast immunophilin (ScFKBP12) that acts redundantly to TWD1 (Henrichs et al., 2012). Taken together, this indicates that PID-dependent phosphorylation of ABCB1 has an activating or inhibiting effect depending on the absence or presence of TWD1 (Figures 3C,D). In this way, TWD1 might control the rate of polar auxin transport, by serving as a switch between asymmetric polar auxin efflux by PIN family transporters and symmetric auxin efflux by ABCB1 (Mravec et al., 2008). Interestingly, the effect of PID-mediated phosphorylation differs per type of transporter: for PIN proteins it directs subcellular localization, whereas for ABCB1 it affects auxin transport activity (Figures 3 and 4).

## Conclusion

Plants share the basic AGC kinase subfamilies (PDK1, S6Ks, and NDRs) with other eukaryotes, but lack the typical PKA and PKC subfamilies that are involved in regulating cell growth, -division, and -polarity in animals and yeast. Instead, plants seem to have adopted the AGCVIII kinase subfamily to perform similar functions. The first AGCVIII kinase appeared in unicellular algae as BL-sensing photoreceptor (phot2), whose signaling protects the cells against high fluency rate BL. During the evolution of multicellular plant life and the move from water to land, the AGCVIII kinases diversified by losing the photo-sensing domain, and were recruited for other functions such as control of auxin transport (PID, WAG1, WAG2, D6PK, and D6PK-L) and pathogen responses (OXI1) (Galvan-Ampudia and Offringa, 2007). Even though the vital role of these kinases for proper plant development is now well-established, not much is known about their regulation and only a few substrates have been identified to date.

The strong conservation of the catalytic kinase core among AGC kinases implies that functional and structural knowledge obtained by detailed studies on mammalian AGC kinases also applies to plant AGC kinases. For example, two basic regulatory processes identified in animal kinases were later also demonstrated for plant AGC kinases: (i) the necessity of T-loop phosphorylation for full activation, and (ii) the potential involvement of PDK1 in this activation, including its interaction via the C-terminal PIF domain. It remains to be determined, however, how plant AGC kinases are activated. With PDK1 a general upstream activator of AGC kinases in plants might be present, but at least for the phototropin kinase domains and several other AGC kinases (PID, WAG1, WAG2, OXI1) auto-activation has been reported. For PID, activation was stronger in the presence of PDK1 (Zegzouti et al., 2006a,b). Other upstream kinases that phosphorylate the T-loop of AGC kinases have not yet been identified.

Next to activation of the T-loop we have highlighted the potential regulatory domains in the insertion domain of the activation segment and in the N- and C-tail that extend from the kinase core of plant AGC kinases. As in animal AGC kinases, the relatively large N- and C-tails of plant AGC kinases suggest that protein–protein interaction domains may be found here. The insertion domain that is typical for the plant specific AGCVIII kinases was found to be essential for proper subcellular localization of *Arabidopsis* PID and tomato Adi3. Although this may only be one of the functional aspects of the insertion domain, it is likely that also other plant AGC kinases have a signaling sequence in their insertion domain that guides them to their proper location. One might assume that the conformation of this signaling sequence changes depending on T-loop phosphorylation status and the interaction with regulatory proteins. This would give the cell a straightforward mechanism to control the localization of activated AGC kinases. Several AGCVIII kinases have been shown to be present at multiple locations within the cell (e.g., phototropins, WAG1, WAG2, ZmBIF2). It will be interesting to see which intrinsic signals instruct their subcellular localization, and whether this is modulated in response to distinct stimuli. Further dissection of the AGC kinase subdomains guided by the structural knowledge presented in this review will help to understand the dynamics of intracellular localization of plant AGC kinases in relation to the phosphorylation state of their targets and the processes that they regulate.

The identification of novel AGC kinases can be surprising as a recent report on *Lotus japonicus* PKL01, an NDR kinase (AGCVII) homolog, describes this AGC kinases as being able to phosphorylate tyrosine residues next to its conventional serine/threonine kinase activity (Katayama et al., 2012). To our knowledge the function of AGCVII kinases in *Arabidopsis* has not been determined yet. It will be interesting to see if this expanded function is present in *Arabidopsis*, and which structural changes might enable the dual-specificity of the NDR kinases.

Furthermore, homologs of *Arabidopsis* AGC kinases have been characterized in other plant species (see Table 2). Next to demonstrating similar roles of some AGC kinases in different plant species, these studies also revealed interesting differential aspects of AGC kinase functionality. For example, in contrast to the reported conserved functions of several AGCVIII kinases, INCOMPLETE ROOT HAIR ELONGATION (IRE) a kinase of the “AGC other” group seems to have acquired a new function in *Medicago truncatula*. In *Arabidopsis* this kinase has been shown to regulate root hair elongation (Oyama et al., 2002), while in *Medicago* a role in the formation of nodules has been described (Pislariu and Dickstein, 2007). Whether this is due to the lack of distinct downstream targets in *Arabidopsis*, or due to a changed role of IRE in *Medicago* remains unknown, and mis-expression experiments with the two proteins as well as identification of substrates in the respective plants will be needed.

**Table 2:** **AGC kinases characterized in other plant species**.

Kinase name	Species	Function	*Arabidopsis* homolog	Literature
Adi3	*Solanum lycopersicum*	Cell death suppression	AGC1–3	Devarenne et al. (2006)
BARREN INFLORESCENCE 2 (BIF2), OsPID, PsPK2	*Zea mays, Oryza sativa, Pisum sativum*	Auxin transport	PID, WAG1, WAG2	Bai et al. (2005), McSteen et al. (2007), Morita and Kyozuka (2007)
IRE	*Medicago truncatula*	Nodule formation	IRE	Pislariu and Dickstein (2007)
PDK1	*Oryza sativa, Physcomitrella patens*	AGC kinase activation	PDK1	Matsui et al. (2010), Dittrich and Devarenne (2012)
Phototropins	*Avena sativa, Chlamydomonas reinhardtii, Oryza sativa, Pisum sativum, Vicia faba*, etc.	Blue light perception	PHOT1/2	Phylogenetic overview in Lariguet and Dunand (2005)
PKV	*Solanum lycopersicum*	Disease resistance	AGC1-7	Hammond and Zhao (2000)
PKL01	*Lotus japonicus*	n. d.	NDR	Kameshita et al. (2010)
OXI1	*Oryza sativa*	Disease resistance	OXI1/AGC2–1	Matsui et al. (2010)
CsPK3, PsPK3	*Cucumis sativus, Pisum sativum*	Auxin transport	WAG1, WAG2	Santner and Watson (2006)

*Homologs of various *Arabidopsis* AGC kinases have been described in several plant species*.

*The table respectively indicates the name of the kinase, the plant species in which it has been identified, the biological process in which the kinase functions, the corresponding *Arabidopsis* homologs, and literature references. Homologs of phototropins have been identified in a plethora of different plant species. For initial reading we refer here to a review. The regulatory function of PKL01 in *Lotus japonicus* has not been determined yet. A recent report indicates that it might be able to phosphorylate tyrosine as well as serine and threonine residues*.

In conclusion, plant AGC kinases are an interesting group of kinases that perform functions conserved in all eukaryotes, but also plant-specific functions. Especially the plant-specific AGCVIII kinases have several interesting features, such as their dynamic subcellular localization, and we are confident that further dissection of their structure and function will lead us to new paradigms in signaling and cell polarity regulation.

## Conflict of Interest Statement

The authors declare that the research was conducted in the absence of any commercial or financial relationships that could be construed as a potential conflict of interest.

## References

[B1] Anthony R. G. Henriques R. Helfer A. Meszaros T. Rios G. Testerink C. (2004). A protein kinase target of a PDK1 signalling pathway is involved in root hair growth in *Arabidopsis*. EMBO J. 23, 572–581 10.1038/sj.emboj.7600068 14749726 PMC1271803

[B2] Babourina O. Newman I. Shabala S. (2002). Blue light-induced kinetics of H+ and Ca2+ fluxes in etiolated wild-type and phototropin-mutant *Arabidopsis* seedlings. Proc. Natl. Acad. Sci. U.S.A. 99, 2433–2438 10.1073/pnas.042294599 11854534 PMC122382

[B3] Bai F. Watson J. C. Walling J. Weeden N. Santner A. A. Demason D. A. (2005). Molecular characterization and expression of *PsPK2*, a *PINOID*-like gene from pea (*Pisum sativum*). Plant Sci. 168, 1281–1291 10.1016/j.plantsci.2005.01.005

[B4] Balendran A. Biondi R. M. Cheung P. C. Casamayor A. Deak M. Alessi D. R. (2000). A 3-phosphoinositide-dependent protein kinase-1 (PDK1) docking site is required for the phosphorylation of protein kinase Czeta (PKCzeta) and PKC-related kinase 2 by PDK1. J. Biol. Chem. 275, 20806–20813 10.1074/jbc.M000421200 10764742

[B5] Bastidas A. C. Deal M. S. Steichen J. M. Keshwani M. M. Guo Y. Taylor S. S. (2012). Role of N-terminal myristylation in the structure and regulation of cAMP-dependent protein kinase. J. Mol. Biol. 422, 215–229 10.1016/j.jmb.2012.05.021 22617327 PMC3597442

[B6] Baum G. Long J. C. Jenkins G. I. Trewavas A. J. (1999). Stimulation of the blue light phototropic receptor NPH1 causes a transient increase in cytosolic Ca2+. Proc. Natl. Acad. Sci. U.S.A. 96, 13554–13559 10.1073/pnas.96.23.13554 10557359 PMC23986

[B7] Benjamins R. Ampudia C. S. Hooykaas P. J. Offringa R. (2003). PINOID-mediated signaling involves calcium-binding proteins. Plant Physiol. 132, 1623–1630 10.1104/pp.103.019943 12857841 PMC167099

[B8] Benjamins R. Quint A. Weijers D. Hooykaas P. Offringa R. (2001). The PINOID protein kinase regulates organ development in *Arabidopsis* by enhancing polar auxin transport. Development 128, 4057–4067 11641228 10.1242/dev.128.20.4057

[B9] Benschop J. J. Mohammed S. O’Flaherty M. Heck A. J. Slijper M. Menke F. L. (2007). Quantitative phosphoproteomics of early elicitor signaling in *Arabidopsis*. Mol. Cell Proteomics 6, 1198–1214 10.1074/mcp.M600429-MCP200 17317660

[B10] Biondi R. M. (2004). Phosphoinositide-dependent protein kinase 1, a sensor of protein conformation. Trends Biochem. Sci. 29, 136–142 10.1016/j.tibs.2004.01.005 15003271

[B11] Biondi R. M. Cheung P. C. Casamayor A. Deak M. Currie R. A. Alessi D. R. (2000). Identification of a pocket in the PDK1 kinase domain that interacts with PIF and the C-terminal residues of PKA. EMBO J. 19, 979–988 10.1093/emboj/19.5.979 10698939 PMC305637

[B12] Biondi R. M. Kieloch A. Currie R. A. Deak M. Alessi D. R. (2001). The PIF-binding pocket in PDK1 is essential for activation of S6K and SGK, but not PKB. EMBO J. 20, 4380–4390 10.1093/emboj/20.16.4380 11500365 PMC125563

[B13] Blanc G. Duncan G. Agarkova I. Borodovsky M. Gurnon J. Kuo A. (2010). The *Chlorella variabilis* NC64A genome reveals adaptation to photosymbiosis, coevolution with viruses, and cryptic sex. Plant Cell 22, 2943–2955 10.1105/tpc.110.076406 20852019 PMC2965543

[B14] Bögre L. Okresz L. Henriques R. Anthony R. G. (2003). Growth signalling pathways in *Arabidopsis* and the AGC protein kinases. Trends Plant Sci. 8, 424–431 10.1016/S1360-1385(03)00188-2 13678909

[B15] Bouchard R. Bailly A. Blakeslee J. J. Oehring S. C. Vincenzetti V. Lee O. R. (2006). Immunophilin-like TWISTED DWARF1 modulates auxin efflux activities of *Arabidopsis* P-glycoproteins. J. Biol. Chem. 281, 30603–30612 10.1074/jbc.M604604200 16887800

[B16] Camehl I. Drzewiecki C. Vadassery J. Shahollari B. Sherameti I. Forzani C. (2011). The OXI1 kinase pathway mediates *Piriformospora indica*-induced growth promotion in *Arabidopsis*. PLoS Pathog. 7, e1002051 10.1371/journal.ppat.1002051 21625539 PMC3098243

[B17] Carafoli E. Santella L. Branca D. Brini M. (2001). Generation, control, and processing of cellular calcium signals. Crit. Rev. Biochem. Mol. Biol. 36, 107–260 10.1080/20014091074183 11370791

[B18] Casamayor A. Morrice N. A. Alessi D. R. (1999). Phosphorylation of Ser-241 is essential for the activity of 3-phosphoinositide-dependent protein kinase-1: identification of five sites of phosphorylation in vivo. Biochem. J. 342(Pt 2), 287–292 10.1042/0264-6021:3420287 10455013 PMC1220463

[B19] Chan T. O. Rittenhouse S. E. Tsichlis P. N. (1999). AKT/PKB and other D3 phosphoinositide-regulated kinases: kinase activation by phosphoinositide-dependent phosphorylation. Annu. Rev. Biochem. 68, 965–1014 10.1146/annurev.biochem.68.1.965 10872470

[B20] Chen Y. Hoehenwarter W. Weckwerth W. (2010). Comparative analysis of phytohormone-responsive phosphoproteins in *Arabidopsis thaliana* using TiO2-phosphopeptide enrichment and mass accuracy precursor alignment. Plant J. 63, 1–17 10.1111/j.1365-313X.2010.04261.x 20374526

[B21] Cheng X. Ma Y. Moore M. Hemmings B. A. Taylor S. S. (1998). Phosphorylation and activation of cAMP-dependent protein kinase by phosphoinositide-dependent protein kinase. Proc. Natl. Acad. Sci. U.S.A. 95, 9849–9854 10.1073/pnas.95.6.3042 9707564 PMC21425

[B22] Cheng Y. Qin G. Dai X. Zhao Y. (2008). *NPY* genes and AGC kinases define two key steps in auxin-mediated organogenesis in *Arabidopsis*. Proc. Natl. Acad. Sci. U.S.A. 105, 21017–21022 10.1073/pnas.0710990105 19075219 PMC2634868

[B23] Christensen S. K. Dagenais N. Chory J. Weigel D. (2000). Regulation of auxin response by the protein kinase PINOID. Cell 100, 469–478 10.1016/S0092-8674(00)80682-0 10693763

[B24] Christie J. M. (2007). Phototropin blue-light receptors. Annu. Rev. Plant Biol. 58, 21–45 10.1146/annurev.arplant.58.032806.103951 17067285

[B25] Christie J. M. Yang H. Richter G. L. Sullivan S. Thomson C. E. Lin J. (2011). phot1 inhibition of ABCB19 primes lateral auxin fluxes in the shoot apex required for phototropism. PLoS Biol. 9, e1001076 10.1371/journal.pbio.1001076 21666806 PMC3110179

[B26] Currie R. A. Walker K. S. Gray A. Deak M. Casamayor A. Downes C. P. (1999). Role of phosphatidylinositol 3,4,5-trisphosphate in regulating the activity and localization of 3-phosphoinositide-dependent protein kinase-1. Biochem. J. 337(Pt 3), 575–583 10.1042/0264-6021:3370575 9895304 PMC1220012

[B27] Dai M. Zhang C. Kania U. Chen F. Xue Q. McCray T. (2012). A PP6-type phosphatase holoenzyme directly regulates PIN phosphorylation and auxin efflux in *Arabidopsis*. Plant Cell. 24, 2497–2514 22715043 10.1105/tpc.112.098905 PMC3406902

[B28] Day I. S. Miller C. Golovkin M. Reddy A. S. (2000). Interaction of a kinesin-like calmodulin-binding protein with a protein kinase. J. Biol. Chem. 275, 13737–13745 10.1074/jbc.275.18.13737 10788494

[B29] Deak M. Casamayor A. Currie R. A. Downes C. P. Alessi D. R. (1999). Characterisation of a plant 3-phosphoinositide-dependent protein kinase-1 homologue which contains a pleckstrin homology domain. FEBS Lett. 451, 220–226 10.1016/S0014-5793(99)00556-6 10371193

[B30] Demarsy E. Schepens I. Okajima K. Hersch M. Bergmann S. Christie J. (2012). Phytochrome Kinase Substrate 4 is phosphorylated by the phototropin 1 photoreceptor. EMBO J. 31, 3457–3467 10.1038/emboj.2012.186 22781128 PMC3419926

[B31] Derelle E. Ferraz C. Rombauts S. Rouze P. Worden A. Z. Robbens S. (2006). Genome analysis of the smallest free-living eukaryote *Ostreococcus tauri* unveils many unique features. Proc. Natl. Acad. Sci. U.S.A. 103, 11647–11652 10.1073/pnas.0604795103 16868079 PMC1544224

[B32] Devarenne T. P. Ekengren S. K. Pedley K. F. Martin G. B. (2006). Adi3 is a PDK1-interacting AGC kinase that negatively regulates plant cell death. EMBO J. 25, 255–265 10.1038/sj.emboj.7600910 16362044 PMC1356353

[B33] Dhonukshe P. Aniento F. Hwang I. Robinson D. G. Mravec J. Stierhof Y. D. (2007). Clathrin-mediated constitutive endocytosis of PIN auxin efflux carriers in *Arabidopsis*. Curr. Biol. 17, 520–527 10.1016/j.cub.2007.01.052 17306539

[B34] Dhonukshe P. Huang F. Galvan-Ampudia C. S. Mahonen A. P. Kleine-Vehn J. Xu J. (2010). Plasma membrane-bound AGC3 kinases phosphorylate PIN auxin carriers at TPRxS(N/S) motifs to direct apical PIN recycling. Development 137, 3245–3255 10.1242/dev.052456 20823065

[B35] Ding Z. Galvan-Ampudia C. S. Demarsy E. Langowski L. Kleine-Vehn J. Fan Y. (2011). Light-mediated polarization of the PIN3 auxin transporter for the phototropic response in *Arabidopsis*. Nat. Cell Biol. 13, 447–452 10.1038/ncb2208 21394084

[B36] Dittrich A. C. Devarenne T. P. (2012). Characterization of a PDK1 homologue from the moss *Physcomitrella patens*. Plant Physiol. 158, 1018–1033 10.1104/pp.111.184572 22158524 PMC3271739

[B37] Ek-Ramos M. J. Avila J. Cheng C. Martin G. B. Devarenne T. P. (2010). The T-loop extension of the tomato protein kinase AvrPto-dependent Pto-interacting protein 3 (Adi3) directs nuclear localization for suppression of plant cell death. J. Biol. Chem. 285, 17584–17594 10.1074/jbc.M110.117416 20371603 PMC2878523

[B38] Enugutti B. Kirchhelle C. Oelschner M. Torres Ruiz R. A. Schliebner I. Leister D. (2012). Regulation of planar growth by the *Arabidopsis* AGC protein kinase UNICORN. Proc. Natl. Acad. Sci. U.S.A. 109, 15060–15065 10.1073/pnas.1205089109 22927420 PMC3443170

[B39] Etchebehere L. C. Van Bemmelen M. X. Anjard C. Traincard F. Assemat K. Reymond C. (1997). The catalytic subunit of *Dictyostelium* cAMP-dependent protein kinase – role of the N-terminal domain and of the C-terminal residues in catalytic activity and stability. Eur. J. Biochem. 248, 820–826 10.1111/j.1432-1033.1997.t01-2-00820.x 9342234

[B40] Folta K. M. Spalding E. P. (2001). Unexpected roles for cryptochrome 2 and phototropin revealed by high-resolution analysis of blue light-mediated hypocotyl growth inhibition. Plant J. 26, 471–478 10.1046/j.1365-313x.2001.01038.x 11439133

[B41] Friml J. Yang X. Michniewicz M. Weijers D. Quint A. Tietz O. (2004). A PINOID-dependent binary switch in apical-basal PIN polar targeting directs auxin efflux. Science 306, 862–865 10.1126/science.1100618 15514156

[B42] Frodin M. Antal T. L. Dummler B. A. Jensen C. J. Deak M. Gammeltoft S. (2002). A phosphoserine/threonine-binding pocket in AGC kinases and PDK1 mediates activation by hydrophobic motif phosphorylation. EMBO J. 21, 5396–5407 10.1093/emboj/cdf551 12374740 PMC129083

[B43] Galvan-Ampudia C. S. Offringa R. (2007). Plant evolution: AGC kinases tell the auxin tale. Trends Plant Sci. 12, 541–547 10.1016/j.tplants.2007.10.004 18024140

[B44] Gao X. Harris T. K. (2006). Role of the PH domain in regulating in vitro autophosphorylation events required for reconstitution of PDK1 catalytic activity. Bioorg. Chem. 34, 200–223 10.1016/j.bioorg.2006.05.002 16780920

[B45] Geldner N. Anders N. Wolters H. Keicher J. Kornberger W. Muller P. (2003). The *Arabidopsis* GNOM ARF-GEF mediates endosomal recycling, auxin transport, and auxin-dependent plant growth. Cell 112, 219–230 10.1016/S0092-8674(03)00003-5 12553910

[B46] Geldner N. Friml J. Stierhof Y. D. Jürgens G. Palme K. (2001). Auxin transport inhibitors block PIN1 cycling and vesicle trafficking. Nature 413, 425–428 10.1038/35096571 11574889

[B47] Gleason C. Chaudhuri S. Yang T. Munoz A. Poovaiah B. W. Oldroyd G. E. (2006). Nodulation independent of rhizobia induced by a calcium-activated kinase lacking autoinhibition. Nature 441, 1149–1152 10.1038/nature04812 16810256

[B48] Hammond R. W. Zhao Y. (2000). Characterization of a tomato protein kinase gene induced by infection by *Potato spindle tuber viroid*. Mol. Plant Microbe Interact. 13, 903–910 10.1094/MPMI.2000.13.9.903 10975647

[B49] Hanks S. K. Hunter T. (1995). Protein kinases 6. The eukaryotic protein kinase superfamily: kinase (catalytic) domain structure and classification. FASEB J. 9, 576–596 7768349

[B50] Harada A. Sakai T. Okada K. (2003). Phot1 and phot2 mediate blue light-induced transient increases in cytosolic Ca2+ differently in *Arabidopsis* leaves. Proc. Natl. Acad. Sci. U.S.A. 100, 8583–8588 10.1073/pnas.0235680100 12821778 PMC166272

[B51] Henrichs S. Wang B. Fukao Y. Zhu J. Charrier L. Bailly A. (2012). Regulation of ABCB1/PGP1-catalysed auxin transport by linker phosphorylation. EMBO J. 31, 2965–2980 10.1038/emboj.2012.120 22549467 PMC3395086

[B52] Hepler P. K. (2005). Calcium: a central regulator of plant growth and development. Plant Cell 17, 2142–2155 10.1105/tpc.105.032508 16061961 PMC1182479

[B53] Hrabak E. M. Chan C. W. Gribskov M. Harper J. F. Choi J. H. Halford N. (2003). The *Arabidopsis* CDPK-SnRK superfamily of protein kinases. Plant Physiol. 132, 666–680 10.1104/pp.102.011999 12805596 PMC167006

[B54] Huang F. Zago M. K. Abas L. Van Marion A. Galvan-Ampudia C. S. Offringa R. (2010). Phosphorylation of conserved PIN motifs directs *Arabidopsis* PIN1 polarity and auxin transport. Plant Cell 22, 1129–1142 10.1105/tpc.109.072678 20407025 PMC2879764

[B55] Huse M. Kuriyan J. (2002). The conformational plasticity of protein kinases. Cell 109, 275–282 10.1016/S0092-8674(02)00741-9 12015977

[B56] Inoue S. Kinoshita T. Matsumoto M. Nakayama K. I. Doi M. Shimazaki K. (2008). Blue light-induced autophosphorylation of phototropin is a primary step for signaling. Proc. Natl. Acad. Sci. U.S.A. 105, 5626–5631 10.1073/pnas.0709189105 18378899 PMC2291087

[B57] Inoue S. Takemiya A. Shimazaki K. (2010). Phototropin signaling and stomatal opening as a model case. Curr. Opin. Plant Biol. 13, 587–593 10.1016/j.pbi.2010.09.002 20920881

[B58] Isner J. C. Nuhse T. Maathuis F. J. (2012). The cyclic nucleotide cGMP is involved in plant hormone signalling and alters phosphorylation of *Arabidopsis thaliana* root proteins. J. Exp. Bot. 63, 3199–3205 10.1093/jxb/ers045 22345640 PMC3350932

[B59] Iwabuchi K. Minamino R. Takagi S. (2010). Actin reorganization underlies phototropin-dependent positioning of nuclei in *Arabidopsis* leaf cells. Plant Physiol. 152, 1309–1319 10.1104/pp.109.149526 20107027 PMC2832274

[B60] Jacobs M. Rubery P. H. (1988). Naturally occurring auxin transport regulators. Science 241, 346–349 10.1126/science.241.4863.346 17734864

[B61] Jiang T. Qiu Y. (2003). Interaction between Src and a C-terminal proline-rich motif of Akt is required for Akt activation. J. Biol. Chem. 278, 15789–15793 10.1074/jbc.M303598200 12600984

[B62] Kaiserli E. Sullivan S. Jones M. A. Feeney K. A. Christie J. M. (2009). Domain swapping to assess the mechanistic basis of *Arabidopsis* phototropin 1 receptor kinase activation and endocytosis by blue light. Plant Cell 21, 3226–3244 10.1105/tpc.109.067876 19880798 PMC2782288

[B63] Kameshita I. Shimomura S. Nishio K. Sueyoshi N. Nishida T. Nomura M. (2010). Expression and characterization of PKL01, an Ndr kinase homolog in *Lotus japonicus*. J. Biochem. 147, 799–807 10.1093/jb/mvq011 20139062

[B64] Kannan N. Haste N. Taylor S. S. Neuwald A. F. (2007). The hallmark of AGC kinase functional divergence is its C-terminal tail, a cis-acting regulatory module. Proc. Natl. Acad. Sci. U.S.A. 104, 1272–1277 10.1073/pnas.0610251104 17227859 PMC1783090

[B65] Katayama S. Sugiyama Y. Hatano N. Terachi T. Sueyoshi N. Kameshita I. (2012). PKL01, an NDR kinase homologue in plants, shows tyrosine kinase activity. J. Biochem. 152, 347–353 10.1093/jb/mvs075 22753892

[B66] Kleine-Vehn J. Huang F. Naramoto S. Zhang J. Michniewicz M. Offringa R. (2009). PIN auxin efflux carrier polarity is regulated by PINOID kinase-mediated recruitment into GNOM-independent trafficking in *Arabidopsis*. Plant Cell 21, 3839–3849 10.1105/tpc.109.071639 20040538 PMC2814515

[B67] Kleine-Vehn J. Langowski L. Wisniewska J. Dhonukshe P. Brewer P. B. Friml J. (2008). Cellular and molecular requirements for polar PIN targeting and transcytosis in plants. Mol Plant 1, 1056–1066 10.1093/mp/ssn062 19825603

[B68] Kleine-Vehn J. Wabnik K. Martiniere A. Langowski L. Willig K. Naramoto S. (2011). Recycling, clustering, and endocytosis jointly maintain PIN auxin carrier polarity at the plasma membrane. Mol. Syst. Biol. 7, 540 10.1038/msb.2011.72 22027551 PMC3261718

[B69] Kong S. G. Suzuki T. Tamura K. Mochizuki N. Hara-Nishimura I. Nagatani A. (2006). Blue light-induced association of phototropin 2 with the Golgi apparatus. Plant J. 45, 994–1005 10.1111/j.1365-313X.2006.02667.x 16507089

[B70] Lariguet P. Dunand C. (2005). Plant photoreceptors: phylogenetic overview. J. Mol. Evol. 61, 559–569 10.1007/s00239-004-0294-2 16170454

[B71] Lariguet P. Schepens I. Hodgson D. Pedmale U. V. Trevisan M. Kami C. (2006). PHYTOCHROME KINASE SUBSTRATE 1 is a phototropin 1 binding protein required for phototropism. Proc. Natl. Acad. Sci. U.S.A. 103, 10134–10139 10.1073/pnas.0603799103 16777956 PMC1502518

[B72] Lewis D. R. Ramirez M. V. Miller N. D. Vallabhaneni P. Ray W. K. Helm R. F. (2011). Auxin and ethylene induce flavonol accumulation through distinct transcriptional networks. Plant Physiol. 156, 144–164 10.1104/pp.111.172502 21427279 PMC3091047

[B73] Li H. Lin D. Dhonukshe P. Nagawa S. Chen D. Friml J. (2011). Phosphorylation switch modulates the interdigitated pattern of PIN1 localization and cell expansion in *Arabidopsis* leaf epidermis. Cell Res. 21, 970–978 10.1038/cr.2011.186 21423279 PMC3203702

[B74] Mahfouz M. M. Kim S. Delauney A. J. Verma D. P. (2006). *Arabidopsis* TARGET OF RAPAMYCIN interacts with RAPTOR, which regulates the activity of S6 kinase in response to osmotic stress signals. Plant Cell 18, 477–490 10.1105/tpc.105.035931 16377759 PMC1356553

[B75] Manning G. Plowman G. D. Hunter T. Sudarsanam S. (2002). Evolution of protein kinase signaling from yeast to man. Trends Biochem. Sci. 27, 514–520 10.1016/S0968-0004(02)02179-5 12368087

[B76] Masters T. A. Calleja V. Armoogum D. A. Marsh R. J. Applebee C. J. Laguerre M. (2010). Regulation of 3-phosphoinositide-dependent protein kinase 1 activity by homodimerization in live cells. Sci. Signal. 3, ra78 10.1126/scisignal.2000738 20978239

[B77] Matsui H. Miyao A. Takahashi A. Hirochika H. (2010). PDK1 kinase regulates basal disease resistance through the OsOxi1-OsPti1a phosphorylation cascade in rice. Plant Cell Physiol. 51, 2082–2091 10.1093/pcp/pcq132 21051443

[B78] McLoughlin F. Galvan-Ampudia C. S. Julkowska M. M. Caarls L. Van Der Does D. Lauriere C. (2012). The Snf1-related protein kinases SnRK2.4 and SnRK2.10 are involved in maintenance of root system architecture during salt stress. Plant J. 72, 436–449 10.1111/j.1365-313X.2012.05089.x 22738204 PMC3533798

[B79] McSteen P. Malcomber S. Skirpan A. Lunde C. Wu X. Kellogg E. (2007). *barren inflorescence2* encodes a co-ortholog of the PINOID serine/threonine kinase and is required for organogenesis during inflorescence and vegetative development in maize. Plant Physiol. 144, 1000–1011 10.1104/pp.107.098558 17449648 PMC1914211

[B80] Men S. Boutte Y. Ikeda Y. Li X. Palme K. Stierhof Y. D. (2008). Sterol-dependent endocytosis mediates post-cytokinetic acquisition of PIN2 auxin efflux carrier polarity. Nat. Cell Biol. 10, 237–244 10.1038/ncb1686 18223643

[B81] Michniewicz M. Zago M. K. Abas L. Weijers D. Schweighofer A. Meskiene I. (2007). Antagonistic regulation of PIN phosphorylation by PP2A and PINOID directs auxin flux. Cell 130, 1044–1056 10.1016/j.cell.2007.07.033 17889649

[B82] Miranda-Saavedra D. Barton G. J. (2007). Classification and functional annotation of eukaryotic protein kinases. Proteins 68, 893–914 10.1002/prot.21444 17557329

[B83] Mora A. Komander D. Van Aalten D. M. Alessi D. R. (2004). PDK1, the master regulator of AGC kinase signal transduction. Semin. Cell Dev. Biol. 15, 161–170 10.1016/j.semcdb.2003.12.022 15209375

[B84] Morita Y. Kyozuka J. (2007). Characterization of OsPID, the rice ortholog of PINOID, and its possible involvement in the control of polar auxin transport. Plant Cell Physiol. 48, 540–549 10.1093/pcp/pcm024 17303594

[B85] Mravec J. Kubes M. Bielach A. Gaykova V. Petrasek J. Skupa P. (2008). Interaction of PIN and PGP transport mechanisms in auxin distribution-dependent development. Development 135, 3345–3354 10.1242/dev.021071 18787070

[B86] Nagawa S. Xu T. Lin D. Dhonukshe P. Zhang X. Friml J. (2012). ROP GTPase-dependent actin microfilaments promote pin1 polarization by localized inhibition of clathrin-dependent endocytosis. PLoS Biol. 10, e1001299 10.1371/journal.pbio.1001299 22509133 PMC3317906

[B87] Newton A. C. (2001). Protein kinase C: structural and spatial regulation by phosphorylation, cofactors, and macromolecular interactions. Chem. Rev. 101, 2353–2364 10.1021/cr0002801 11749377

[B88] Nirula A. Ho M. Phee H. Roose J. Weiss A. (2006). Phosphoinositide-dependent kinase 1 targets protein kinase A in a pathway that regulates interleukin 4. J. Exp. Med. 203, 1733–1744 10.1084/jem.20051715 16785309 PMC2118337

[B89] Nolen B. Taylor S. Ghosh G. (2004). Regulation of protein kinases; controlling activity through activation segment conformation. Mol. Cell 15, 661–675 10.1016/j.molcel.2004.08.024 15350212

[B90] Nuhse T. S. Stensballe A. Jensen O. N. Peck S. C. (2004). Phosphoproteomics of the *Arabidopsis* plasma membrane and a new phosphorylation site database. Plant Cell 16, 2394–2405 10.1105/tpc.104.023150 15308754 PMC520941

[B91] Onodera A. Kong S. G. Doi M. Shimazaki K. Christie J. Mochizuki N. (2005). Phototropin from *Chlamydomonas reinhardtii* is functional in *Arabidopsis thaliana*. Plant Cell Physiol. 46, 367–374 10.1093/pcp/pci037 15695460

[B92] Otterhag L. Gustavsson N. Alsterfjord M. Pical C. Lehrach H. Gobom J. (2006). *Arabidopsis* PDK1: identification of sites important for activity and downstream phosphorylation of S6 kinase. Biochimie 88, 11–21 10.1016/j.biochi.2005.07.005 16125835

[B93] Oyama T. Shimura Y. Okada K. (2002). The *IRE* gene encodes a protein kinase homologue and modulates root hair growth in *Arabidopsis*. Plant J. 30, 289–299 10.1046/j.1365-313X.2002.01290.x 12000677

[B94] Pearce L. R. Komander D. Alessi D. R. (2010). The nuts and bolts of AGC protein kinases. Nat. Rev. Mol. Cell Biol. 11, 9–22 10.1038/nrg2695 20027184

[B95] Pedmale U. V. Liscum E. (2007). Regulation of phototropic signaling in *Arabidopsis* via phosphorylation state changes in the phototropin 1-interacting protein NPH3. J. Biol. Chem. 282, 19992–20001 10.1074/jbc.M702551200 17493935

[B96] Petersen L. N. Ingle R. A. Knight M. R. Denby K. J. (2009). OXI1 protein kinase is required for plant immunity against *Pseudomonas syringae* in *Arabidopsis*. J. Exp. Bot. 60, 3727–3735 10.1093/jxb/erp219 19574254 PMC2736892

[B97] Pfeifer A. Mathes T. Lu Y. Hegemann P. Kottke T. (2010). Blue light induces global and localized conformational changes in the kinase domain of full-length phototropin. Biochemistry 49, 1024–1032 10.1021/bi9016044 20052995

[B98] Pislariu C. I. Dickstein R. (2007). An IRE-like AGC kinase gene, *MtIRE*, has unique expression in the invasion zone of developing root nodules in *Medicago truncatula*. Plant Physiol. 144, 682–694 10.1104/pp.106.092494 17237187 PMC1914176

[B99] Rentel M. C. Lecourieux D. Ouaked F. Usher S. L. Petersen L. Okamoto H. (2004). OXI1 kinase is necessary for oxidative burst-mediated signalling in *Arabidopsis*. Nature 427, 858–861 10.1038/nature02353 14985766

[B100] Roberts D. Pedmale U. V. Morrow J. Sachdev S. Lechner E. Tang X. (2011). Modulation of phototropic responsiveness in *Arabidopsis* through ubiquitination of phototropin 1 by the CUL3-Ring E3 ubiquitin ligase CRL3(NPH3). Plant Cell 23, 3627–3640 10.1105/tpc.111.087999 21990941 PMC3229139

[B101] Sakamoto K. Briggs W. R. (2002). Cellular and subcellular localization of phototropin 1. Plant Cell 14, 1723–1735 10.1105/tpc.003293 12172018 PMC151461

[B102] Salomon M. Christie J. M. Knieb E. Lempert U. Briggs W. R. (2000). Photochemical and mutational analysis of the FMN-binding domains of the plant blue light receptor, phototropin. Biochemistry 39, 9401–9410 10.1021/bi000585+ 10924135

[B103] Salomon M. Knieb E. Von Zeppelin T. Rudiger W. (2003). Mapping of low- and high-fluence autophosphorylation sites in phototropin 1. Biochemistry 42, 4217–4225 10.1021/bi027324f 12680776

[B104] Santner A. A. Watson J. C. (2006). The WAG1 and WAG2 protein kinases negatively regulate root waving in *Arabidopsis*. Plant J. 45, 752–764 10.1111/j.1365-313X.2005.02641.x 16460509

[B105] Sastri M. Barraclough D. M. Carmichael P. T. Taylor S. S. (2005). A kinase-interacting protein localizes protein kinase A in the nucleus. Proc. Natl. Acad. Sci. U.S.A. 102, 349–354 10.1073/pnas.0408608102 15630084 PMC544310

[B106] Sathyanarayanan P. V. Cremo C. R. Poovaiah B. W. (2000). Plant chimeric Ca2+ Calmodulin-dependent protein kinase. Role of the neural visinin-like domain in regulating autophosphorylation and calmodulin affinity. J. Biol. Chem. 275, 30417–30422 10.1074/jbc.M000771200 10840028

[B107] Sieburth L. E. Muday G. K. King E. J. Benton G. Kim S. Metcalf K. E. (2006). SCARFACE encodes an ARF-GAP that is required for normal auxin efflux and vein patterning in *Arabidopsis*. Plant Cell 18, 1396–1411 10.1105/tpc.105.039008 16698946 PMC1475492

[B108] Skirpan A. Culler A. H. Gallavotti A. Jackson D. Cohen J. D. McSteen P. (2009). BARREN INFLORESCENCE2 interaction with ZmPIN1a suggests a role in auxin transport during maize inflorescence development. Plant Cell Physiol. 50, 652–657 10.1093/pcp/pcp006 19153156

[B109] Skirpan A. Wu X. McSteen P. (2008). Genetic and physical interaction suggest that BARREN STALK 1 is a target of BARREN INFLORESCENCE2 in maize inflorescence development. Plant J. 55, 787–797 10.1111/j.1365-313X.2008.03546.x 18466309

[B110] Sukumar P. Edwards K. S. Rahman A. Delong A. Muday G. K. (2009). PINOID kinase regulates root gravitropism through modulation of PIN2-dependent basipetal auxin transport in *Arabidopsis*. Plant Physiol. 150, 722–735 10.1104/pp.108.131607 19363095 PMC2689958

[B111] Sullivan S. Thomson C. E. Kaiserli E. Christie J. M. (2009). Interaction specificity of *Arabidopsis* 14-3-3 proteins with phototropin receptor kinases. FEBS Lett. 583, 2187–2193 10.1016/j.febslet.2009.06.011 19524572

[B112] Swainsbury D. J. Zhou L. Oldroyd G. E. Bornemann S. (2012). Calcium ion binding properties of *Medicago truncatula* calcium/calmodulin-dependent protein kinase. Biochemistry 51, 6895–6907 10.1021/bi300826m 22889004

[B113] Swartz T. E. Corchnoy S. B. Christie J. M. Lewis J. W. Szundi I. Briggs W. R. (2001). The photocycle of a flavin-binding domain of the blue light photoreceptor phototropin. J. Biol. Chem. 276, 36493–36500 10.1074/jbc.M103114200 11443119

[B114] Takayama Y. Nakasako M. Okajima K. Iwata A. Kashojiya S. Matsui Y. (2011). Light-induced movement of the LOV2 domain in an Asp720Asn mutant LOV2-kinase fragment of *Arabidopsis* phototropin 2. Biochemistry 50, 1174–1183 10.1021/bi101689b 21222437

[B115] Taylor S. S. Kornev A. P. (2011). Protein kinases: evolution of dynamic regulatory proteins. Trends Biochem. Sci. 36, 65–77 10.1016/j.tibs.2010.09.006 20971646 PMC3084033

[B116] Tholey A. Pipkorn R. Bossemeyer D. Kinzel V. Reed J. (2001). Influence of myristoylation, phosphorylation, and deamidation on the structural behavior of the N-terminus of the catalytic subunit of cAMP-dependent protein kinase. Biochemistry 40, 225–231 10.1021/bi0021277 11141074

[B117] Tseng T. S. Whippo C. Hangarter R. P. Briggs W. R. (2012). The role of a 14-3-3 protein in stomatal opening mediated by PHOT2 in *Arabidopsis*. Plant Cell 24, 1114–1126 10.1105/tpc.111.092130 22408078 PMC3336120

[B118] Wick M. J. Ramos F. J. Chen H. Quon M. J. Dong L. Q. Liu F. (2003). Mouse 3-phosphoinositide-dependent protein kinase-1 undergoes dimerization and trans-phosphorylation in the activation loop. J. Biol. Chem. 278, 42913–42919 10.1074/jbc.M208518200 12923190

[B119] Xiong Y. Sheen J. (2012). Rapamycin and glucose-target of rapamycin (TOR) protein signaling in plants. J. Biol. Chem. 287, 2836–2842 10.1074/jbc.M112.375493 22134914 PMC3268441

[B120] Xu J. Scheres B. (2005). Dissection of *Arabidopsis* ADP-RIBOSYLATION FACTOR 1 function in epidermal cell polarity. Plant Cell 17, 525–536 10.1105/tpc.104.028449 15659621 PMC548823

[B121] Xu T. Wen M. Nagawa S. Fu Y. Chen J. G. Wu M. J. (2010). Cell surface- and rho GTPase-based auxin signaling controls cellular interdigitation in *Arabidopsis*. Cell 143, 99–110 10.1016/j.cell.2010.09.003 20887895 PMC2950838

[B122] Yonemoto W. McGlone M. L. Grant B. Taylor S. S. (1997). Autophosphorylation of the catalytic subunit of cAMP-dependent protein kinase in *Escherichia coli*. Protein Eng. 10, 915–925 10.1093/protein/10.8.915 9415441

[B123] Zegzouti H. Anthony R. G. Jahchan N. Bögre L. Christensen S. K. (2006a). Phosphorylation and activation of PINOID by the phospholipid signaling kinase 3-phosphoinositide-dependent protein kinase 1 (PDK1) in *Arabidopsis*. Proc. Natl. Acad. Sci. U.S.A. 103, 6404–6409 10.1073/pnas.0510283103 16601102 PMC1458890

[B124] Zegzouti H. Li W. Lorenz T. C. Xie M. Payne C. T. Smith K. (2006b). Structural and functional insights into the regulation of *Arabidopsis* AGC VIIIa kinases. J. Biol. Chem. 281, 35520–35530 10.1074/jbc.M605167200 16973627

[B125] Zhang J. Nodzynski T. Pencik A. Rolcik J. Friml J. (2010). PIN phosphorylation is sufficient to mediate PIN polarity and direct auxin transport. Proc. Natl. Acad. Sci. U.S.A. 107, 918–922 10.1073/pnas.0914991107 20080776 PMC2818920

[B126] Zhang Y. He J. McCormick S. (2009). Two *Arabidopsis* AGC kinases are critical for the polarized growth of pollen tubes. Plant J. 58, 474–484 10.1111/j.1365-313X.2009.03803.x 19144004

[B127] Zhang Y. McCormick S. (2009). AGCVIII kinases: at the crossroads of cellular signaling. Trends Plant Sci. 14, 689–695 10.1016/j.tplants.2009.09.006 19818674

[B128] Zourelidou M. Muller I. Willige B. C. Nill C. Jikumaru Y. Li H. (2009). The polarly localized D6 PROTEIN KINASE is required for efficient auxin transport in *Arabidopsis thaliana*. Development 136, 627–636 10.1242/dev.028365 19168677

